# Supplementary material for: The ESCRT machinery counteracts Nesprin-2G-mediated mechanical forces during nuclear envelope repair
Source: Dev Cell. 2021 Dec 6;56(23):3192–3202.e8. doi: 10.1016/j.devcel.2021.10.022 (PMC8657813; doi:10.1016/j.devcel.2021.10.022)
Supplement: Document S1. Figures S1–S4 [file mmc1.pdf]

Developmental Cell, Volume *56*

Supplemental information

The ESCRT machinery counteracts Nesprin-2G-mediated mechanical forces during nuclear envelope repair

Samuel S. Wallis, Leandro N. Ventimiglia, Evita Otigbah, Elvira Infante, Miguel Angel Cuesta-Geijo, Gururaj Rao Kidiyoor, M. Alejandra Carbajal, Roland A. Fleck, Marco Foiani, Sergi Garcia-Manyes, Juan Martin-Serrano, and Monica Agromayor

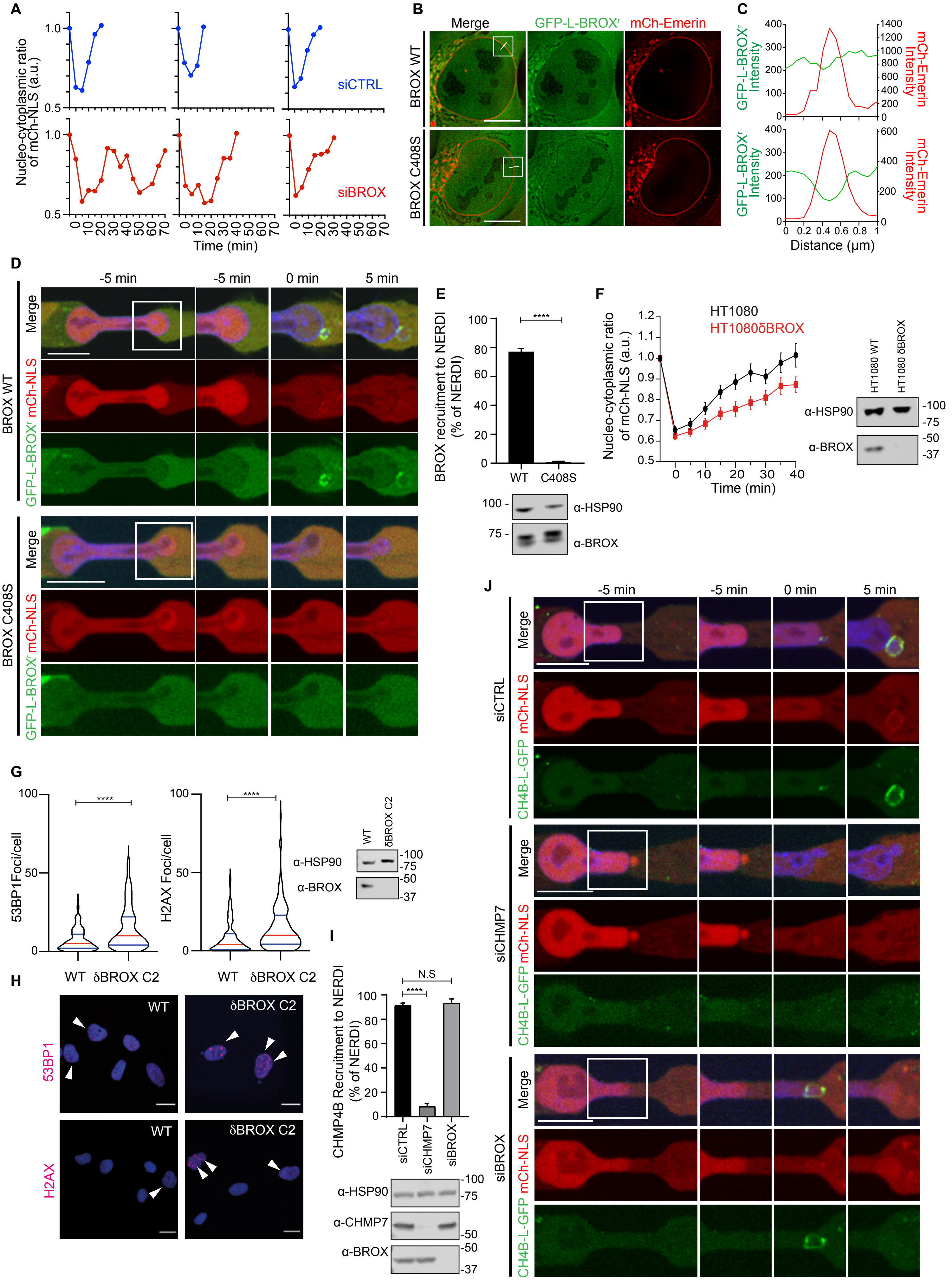
A
Nucleo-cytoplasmic ratio of mCh-NLS (a.u.)
siCTRL
siBROX
Time (min)
B
Merge
GFP-L-BROXʳ
mCh-Emerin
BROX WT
BROX C408S
C
GFP-L-BROXʳ Intensity
mCh-Emerin Intensity
Distance (μm)
D
-5 min
-5 min
0 min
5 min
BROX WT
BROX C408S
Merge
mCh-NLS
GFP-L-BROXʳ
E
BROX recruitment to NERDI (% of NERDI)
****
WT
C408S
α-HSP90
α-BROX
F
Nucleo-cytoplasmic ratio of mCh-NLS (a.u.)
HT1080
HT1080δBROX
Time (min)
HT1080 WT
HT1080 δBROX
α-HSP90
α-BROX
G
53BP1Foci/cell
H2AX Foci/cell
****
WT
δBROX C2
α-HSP90
α-BROX
H
53BP1
H2AX
WT
δBROX C2
I
CHMP4B Recruitment to NERDI (% of NERDI)
N.S
****
siCTRL
siCHMP7
siBROX
α-HSP90
α-CHMP7
α-BROX
J
-5 min
-5 min
0 min
5 min
siCTRL
siCHMP7
siBROX
Merge
mCh-NLS
CH4B-L-GFP

Figure S1: BROX requires farnesylation for NE localisation and does not recruit CHMP4B to NE rupture sites. Related to Figure 1.

(A) Trace analysis of representative individual ruptures of similar size from siCTRL and siBROX treated cells and included in Figure1A.

(**B-C**) Analysis of BROX localisation in live HT1080 cells expressing mCherry-Emerin and GFP-L-BROX^r wild-type (WT) or non-farnesylated mutant (C408S) (B) Deconvolved images corresponding to the quantifications shown in (C). White lines mark the region of the NE where the fluorescence intensity profile was quantified. Scale bar, 10μm. (C) Fluorescence intensity profiles of HT1080 GFP-L-BROX^r WT (top) or C408S (bottom) cells.

(D-E) Time-lapse analysis of HT1080 cells expressing mCherry-NLS and GFP-L-BROX^r WT or C408S migrating through 4μm constrictions after treatment with siBROX. Hoechst was added to stain DNA. (D) Representative images of the quantifications shown in (E). Scale bar, 10μm. (E) Percentage of BROX-decorated NERDIs. GFP-L-BROX^r WT n=80; GFP-L-BROX^r C408S n=81; $p<0.0001$. Cell lysates were examined by blotting with the indicated antibodies.

(F) Recovery kinetics of NE integrity after NERDI in HT1080 wild-type (WT; n=29) or BROX-depleted (δBROX; n=46) cells stably expressing mCherry-NLS. Cell lysates were examined by blotting with the indicated antibodies.

(G-H) Analysis of DNA damage in HT1080 wild-type (WT) and BROX-depleted clone2 (δBROX C2) cells stained with α-53BP1 or α–γH2AX antibodies and Hoechst. (G) Quantification of 53BP1 and γH2AX -positive foci. The samples used for HT1080 WT were the same as in Figure 1I. WT n=147; HT1080$^{\delta BROX}$C2 n=147; $p<0.0001$. Median and quartiles are shown in red and blue respectively and cell lysates were examined by blotting with the indicated antibodies. (H) Representative images of the quantifications shown in (G). Arrowheads show DNA damage foci. Scale bar, 15μm.

(I-J) Time-lapse analysis of HT1080 cells expressing mCherry-NLS and CHMP4B-L-GFP. Cells were transfected with indicated siRNAs and imaged while migrating through 4μm constrictions. Hoechst was added to stain DNA. (I) Percentage of CHMP4B-decorated NERDI. siCTRL n=238; siCHMP7 n=81; siBROX n=137; ****$p<0.0001$, $^{N.S}p>0.05$. Cell lysates were analysed by blotting with the indicated antibodies. (J) Representative images of the quantification shown in (I). Scale bar, 10μm.

Figure S2

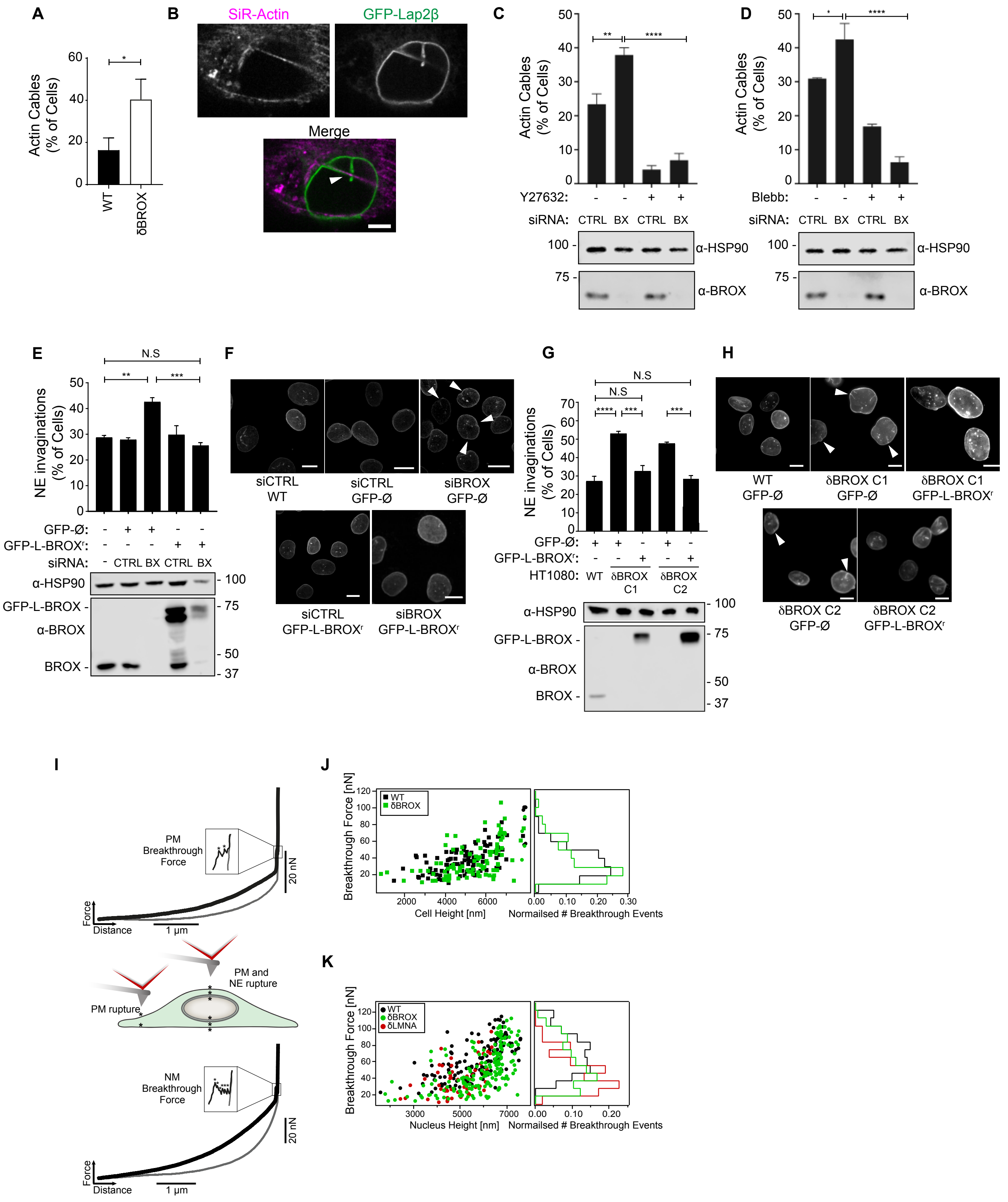

A
Actin Cables (% of Cells)
WT
δBROX
B
SiR-Actin
GFP-Lap2β
Merge
C
Actin Cables (% of Cells)
Y27632:
siRNA: CTRL BX CTRL BX
α-HSP90
α-BROX
D
Actin Cables (% of Cells)
Blebb:
siRNA: CTRL BX CTRL BX
α-HSP90
α-BROX
E
N.S
NE invaginations (% of Cells)
GFP-Ø:
GFP-L-BROXʳ:
siRNA: CTRL BX CTRL BX
α-HSP90
GFP-L-BROX
α-BROX
BROX
F
siCTRL WT
siCTRL GFP-Ø
siBROX GFP-Ø
siCTRL GFP-L-BROXʳ
siBROX GFP-L-BROXʳ
G
N.S
NE invaginations (% of Cells)
GFP-Ø:
GFP-L-BROXʳ:
HT1080: WT δBROX C1 δBROX C2
α-HSP90
GFP-L-BROX
α-BROX
BROX
H
WT GFP-Ø
δBROX C1 GFP-Ø
δBROX C1 GFP-L-BROXʳ
δBROX C2 GFP-Ø
δBROX C2 GFP-L-BROXʳ
I
PM Breakthrough Force
20 nN
Force
Distance
1 µm
PM and NE rupture
PM rupture
NM Breakthrough Force
20 nN
Force
Distance
1 µm
J
Breakthrough Force [nN]
WT
δBROX
Cell Height [nm]
Normalised # Breakthrough Events
K
Breakthrough Force [nN]
WT
δBROX
δLMNA
Nucleus Height [nm]
Normalised # Breakthrough Events

Figure S2: NE phenotypes resulting from BROX depletion can be rescued by re-expression of GFP-L-BROX^r. Related to Figure 2.

(**A-D**) Analysis of actin cables in HT1080 wild-type (WT) or BROX-depleted (δBROX) stably expressing GFP-Lap2β and stained with SiR-actin. (A) Percentage of cells with nuclear actin cables. WT: n=160; δBROX n=116; p=0.0186. (B) Representative images of the quantifications shown in (A). Arrowhead denotes an actin cable. Scale bar, 5µm. (C-D) Percentage of cells with nuclear actin cables in cells treated with Y27632 or blebbistatin. siCTRL+H_2O: n=675; siBROX+H_2O n=611; siCTRL+Y27632: n=577; siBROX+Y27632 n=576; siCTRL+DMSO: n=680; siBROX+DMSO n=670; siCTRL+Blebb: n=570; siBROX+Blebb n=656; *p=0.0464, **p= 0.0068, ****p<0.0001

(**E-F**) Analysis of nuclear morphology in HT1080 cells stably expressing either GFP (GFP-ø) or GFP-L-BROX^r after treatment with the indicated siRNAs and staining with α-Lamin B1 antibody. (E) Quantification of percentage of cells with NE invaginations. Non-transfected n=559; GFP-ø+siCTRL n=487; GFP-ø+siBROX n=523; GFP-L-BROX^r+siCTRL n=667; GFP-L-BROX^r+siBROX n=721; **p=0.0031, ***p=0.0007, $^{N.S}$p>0.05. Cell lysates were analysed by blotting with the indicated antibodies. (F) Representative images of quantifications shown in (E). Arrowheads indicate NE invaginations. Scale bars, 10µm.

(**G-H**) Analysis of nuclear morphology in HT1080 WT and BROX-depleted cells (δBROX C1 and δBROX C2) stably expressing either GFP-ø or GFP-L-BROX^r after staining with α-Lamin B1 antibody. (G) Quantification of percentage of cells with NE invaginations. WT+GFP-ø n=246; δBROX C1+GFP-ø n=283; δBROX C1+GFP-L-BROX^r n=199, δBROX C2+GFP-ø n=291; δBROX C2+GFP-L-BROX^r n=270; ****p<0.0001, ***p<0.001, $^{N.S}$p>0.05. Cell lysates were analysed by blotting with the indicated antibodies. (H) Representative images of quantifications shown in (G). Arrowheads indicate nuclear NE invaginations. Scale bar, 10µm.

(I) Typical force-extension curves conducted on a live cell when only the plasma membrane (PM; top) or also the nuclear membrane (NM; bottom) are indented. Rupture of the plasma membrane (top inset) is hallmarked by two consecutive discontinuities in the contact region of the force-extension trace, while NE rupture can be fingerprinted by the presence of up to six discontinuities, corresponding to the rupture of the plasma membrane (two) in addition to the four nuclear membranes (bottom inset).

(J-K) Scatterplot of the breakthrough force versus the cell height in the PM or the NM. Histograms display the distribution of breakthrough forces corresponding to the rupture of the PM (J) or the NM (K). The colour-code corresponds to the type of cell analysed: WT (black), δBROX (green) and δLMNA (red). Number of traces are the same as in Figure 2I.

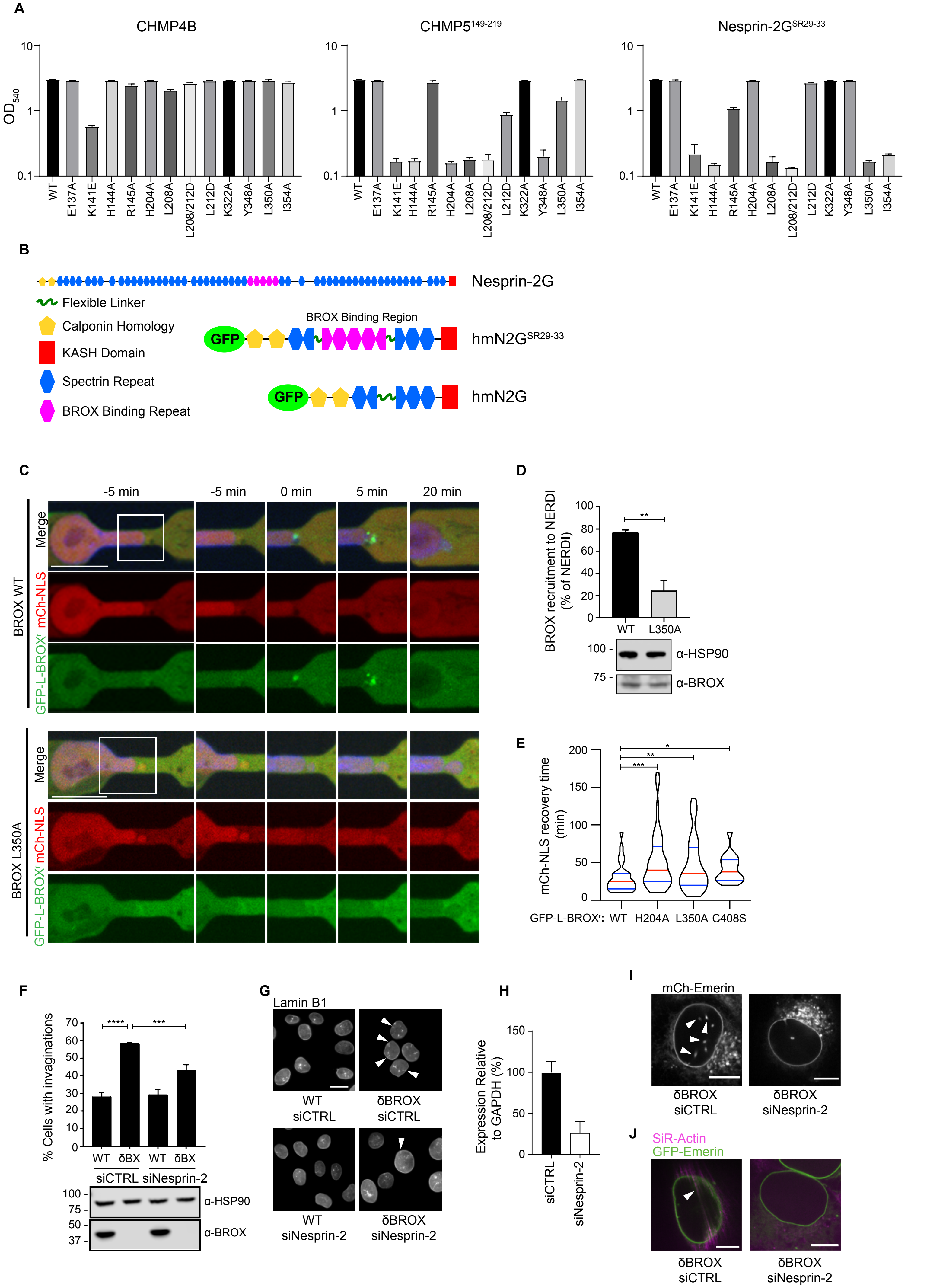
A
CHMP4B
CHMP5149-219
Nesprin-2GSR29-33
OD540
10
1
0.1
WT
E137A
K141E
H144A
R145A
H204A
L208A
L208/212D
L212D
K322A
Y348A
L350A
I354A
B
Nesprin-2G
Flexible Linker
Calponin Homology
KASH Domain
Spectrin Repeat
BROX Binding Repeat
BROX Binding Region
GFP
hmN2GSR29-33
hmN2G
C
-5 min
0 min
5 min
20 min
Merge
mCh-NLS
GFP-L-BROXr
BROX WT
BROX L350A
D
BROX recruitment to NERDI (% of NERDI)
WT
L350A
α-HSP90
α-BROX
E
mCh-NLS recovery time (min)
GFP-L-BROXr: WT H204A L350A C408S
F
% Cells with invaginations
WT δBX WT δBX
siCTRL siNesprin-2
α-HSP90
α-BROX
G
Lamin B1
WT siCTRL
δBROX siCTRL
WT siNesprin-2
δBROX siNesprin-2
H
Expression Relative to GAPDH (%)
siCTRL
siNesprin-2
I
mCh-Emerin
δBROX siCTRL
δBROX siNesprin-2
J
SiR-Actin
GFP-Emerin
δBROX siCTRL
δBROX siNesprin-2

Figure S3: Characterisation of BROX interactions and dependence on Nesprin-2G interaction for localisation during NERDI. Related to Figure 3.

(A) BROX was tested for interactions with CHMP4B (left graph), $CHMP5^{149-219}$ (middle graph) or Nesprin-$2G^{SR29-33}$ (right graph) by yeast two-hybrid assays.

(B) Diagram schematic of human mini-Nesprin-2G (hmN2G) constructs used. hmN2G is based on the previously published mN2G but uses the human sequence of Nesprin-2G. $hmN2GSR^{29-33}$ contains the BROX interacting region identified in the yeast-two hybrid screen flanked by flexible linkers.

(C-D) Time-lapse analysis of HT1080 cells expressing mCherry-NLS and GFP-L-$BROX^r$ wild-type (WT) or L350A mutant cells migrating through 4µm constrictions. Hoechst was added to stain DNA. (C) Representative images of quantifications shown in (D). Scale bar, 10µm. (D) Percentage of BROX-decorated NERDI. GFP-L-$BROX^r$ WT n=80; GFP-L-$BROX^r$ L350A n=113; *p=0.0058. The dataset corresponding to GFP-L-$BROX^r$ WT is the same used in the quantification shown in Figure S1E. Cell lysates were examined by blotting with the indicated antibodies.

(**E**) Quantification of the rupture recovery times in HT1080 cells expressing mCherry-NLS and GFP-L-$BROX^r$ wild-type (WT) or mutant forms (H204A, L350A, C408S); ***P=0.0007, **P=0.0067, *P=0.0391.

(F-G) Analysis of nuclear morphology in HT1080 wild-type (WT) or BROX-depleted (δBROX) cells transfected with indicated siRNAs and stained with α-Lamin B1 antibody. (F) Percentage of cells with NE invaginations. WT+siCTRL n=245; WT+siNesprin-2 n=168; δBROX+siCTRL n=176; δBROX+siNesprin-2 n=193; ****P<0.0001, ***P<0.001. Cell lysates were analysed by blotting with the indicated antibodies. (G) Representative images of quantifications shown in (F). Arrowheads indicate NE invaginations. Scale bar, 20µm.

(H) HT1080 cells transfected with either control or Nesprin-2-specific siRNA were analysed for Nesprin-2 mRNA levels by qPCR. Results were normalised to GAPDH mRNA levels.

(I) Representative images of quantifications in Figure 3H. Arrowheads indicate INTs. Scale bar, 10µm.

(J) Representative images of quantifications in Figure 3I. Arrowhead indicates an actin cable. Scale bar, 10µm.

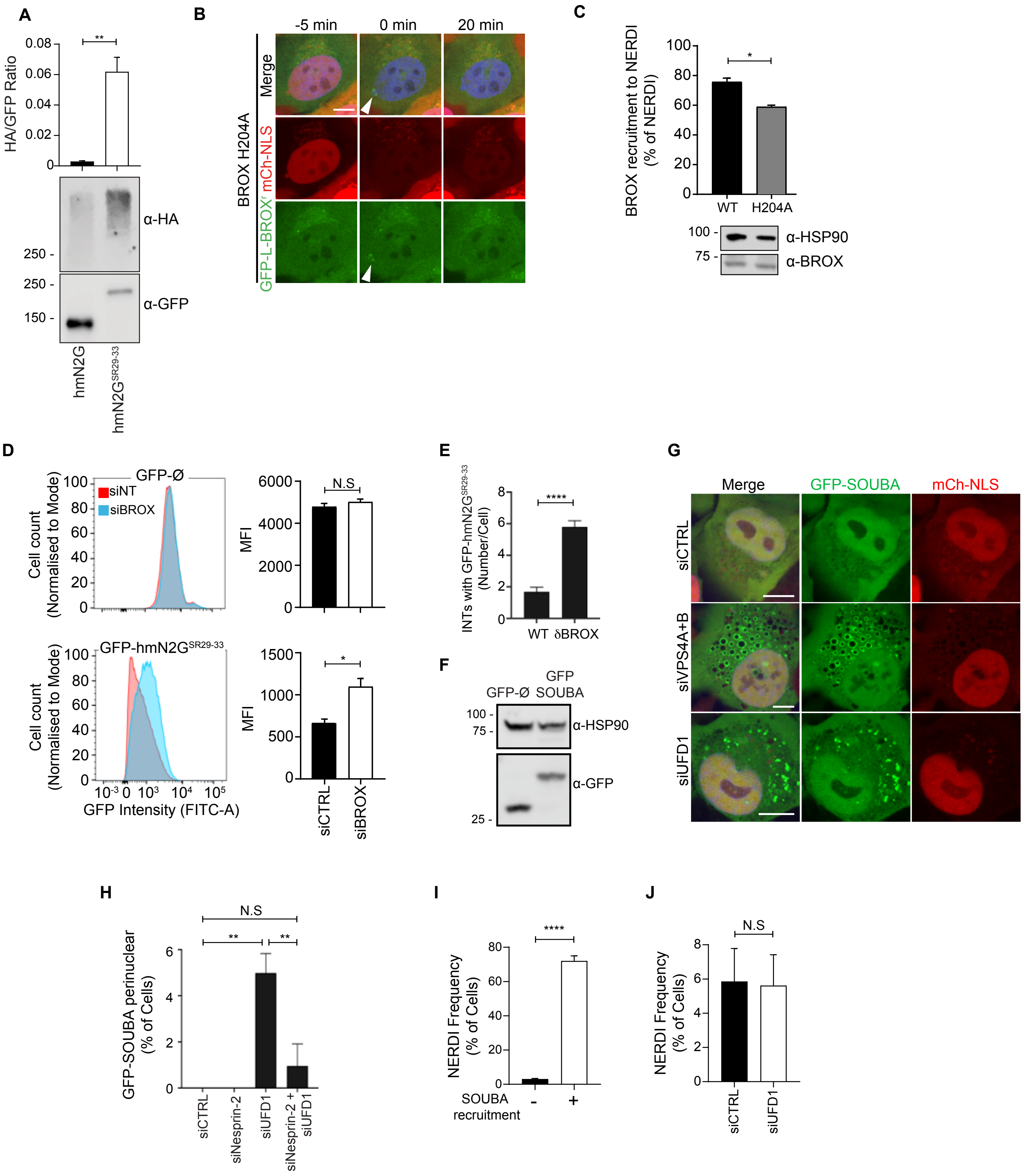

A
HA/GFP Ratio
**
α-HA
α-GFP
250 -
150 -
hmN2G
hmN2G$^{SR29-33}$
B
-5 min
0 min
20 min
Merge
BROX H204A
GFP-L-BROX^{r} mCh-NLS
C
BROX recruitment to NERDI (% of NERDI)
*
WT
H204A
100 -
75 -
α-HSP90
α-BROX
D
GFP-Ø
siNT
siBROX
Cell count (Normalised to Mode)
MFI
N.S
GFP-hmN2G$^{SR29-33}$
GFP Intensity (FITC-A)
siCTRL
siBROX
E
INTs with GFP-hmN2G$^{SR29-33}$ (Number/Cell)
****
WT
δBROX
F
GFP-Ø
GFP SOUBA
α-HSP90
α-GFP
G
Merge
GFP-SOUBA
mCh-NLS
siCTRL
siVPS4A+B
siUFD1
H
GFP-SOUBA perinuclear (% of Cells)
N.S
siCTRL
siNesprin-2
siUFD1
siNesprin-2 + siUFD1
I
NERDI Frequency (% of Cells)
SOUBA recruitment
J
NERDI Frequency (% of Cells)
siCTRL
siUFD1

Figure S4: BROX-CHMP5 interaction is not required for BROX recruitment to rupture sites and GFP-SOUBA perinuclear rings form in cells that will undergo NERDI. Related to Figure 4.

(A) GFP pull-down experiments of 293T transiently co-expressing HA-Ubiquitin together with GFP-hmN2G$^{SR29-33}$ or GFP-hmN2G. Eluted fractions were examined by Western blot using the indicated antibodies and the ratio between HA and GFP bands intensity in the eluted fractions was plotted. p=0.0036

(B-C) Time-lapse analysis of HT1080 cells expressing mCherry-NLS and GFP-L-BROX$^{r.}$ wild-type (WT) or mutant (H204). Hoechst was added to stain DNA. (B) Representative images of the quantifications shown in (C). Scale bar, 5µm. Arrowheads highlight site of rupture (C) Percentage of BROX-decorated NERDI. WT n=38; H204A n=59 cells; p=0.0244. The movies used to quantify recruitment for WT conditions were the same as the ones used for the recruitment quantification shown in Figure 3D. Cell lysates were examined by blotting with the indicated antibodies.

(D) FACS analysis of HT1080 cells stably expressing GFP-empty (GFP-ø) or GFP-hmN2G$^{SR29-33}$ after transfection with indicated siRNAs. Left: GFP fluorescence intensity profiles corresponding to one representative experiment. Right: quantification of GFP mean fluorescence intensity (MFI). *p<0.05, $^{N.S}$p>0.05.

(E) Quantitation of the number of INTs decorated with Nesprin-2G per cell in HT1080 and HT1080$^{\delta BROX}$ cells stably expressing GFP-hmN2G$^{SR29-33}$. WT n=16; δBROX n=10; p<0.0001.

(F) Analysis of GFP-SOUBA expression in HT1080 cells stably expressing GFP-ø or GFP-SOUBA. Cell lysates were analysed by blotting with indicated antibodies.

(G-J) Live imaging of HT1080 cells co-expressing GFP-SOUBA and mCherry-NLS. (G) Analysis of GFP-SOUBA localisation in cells transfected with the indicated siRNAs. Scale bars, 10µm. (H) percentage of cells with perinuclear GFP-SOUBA accumulation. siCTRL n=588; siNesprin2 n=437; siUFD1 n=504; siNesprin2+siUFD1 n=468; **p=0.025, $^{N.S}$p>0.05. (I) Analysis of GFP-SOUBA perinuclear accumulation in cells undergoing NERDI. SOUBA-negative n=1857; SOUBA-positive n=88; p<0.0001. (J) Percentage of cells showing NERDI after transfection with indicated siRNA. siCTRL n=2016; siUFD1 n=1910, p=0.9309.
