# Supplementary material for: The ESCRT machinery counteracts Nesprin-2G-mediated mechanical forces during nuclear envelope repair
Source: Dev Cell. 2021 Dec 6;56(23):3192–3202.e8. doi: 10.1016/j.devcel.2021.10.022 (PMC8657813; doi:10.1016/j.devcel.2021.10.022)
Supplement: Document S2. Article plus supplemental information [file mmc10.pdf]

Short Article

Developmental Cell

The ESCRT machinery counteracts Nesprin-2G-mediated mechanical forces during nuclear envelope repair

Graphical abstract

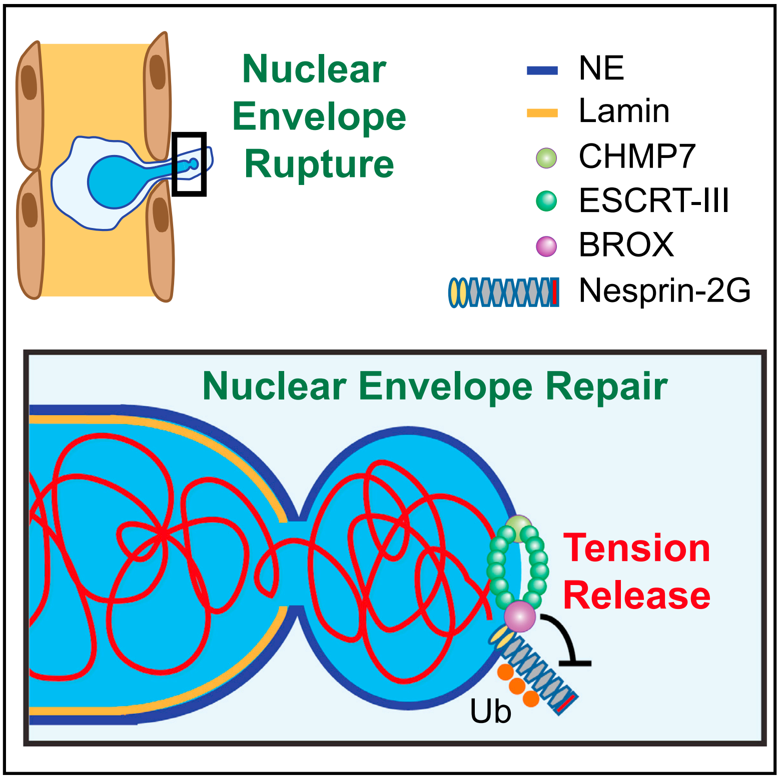

Highlights

- Cytoskeletal forces exerted on the nucleus can rupture its membrane
- BROX is recruited to sites of rupture by the ESCRT membrane remodeling machinery
- BROX ubiquitinates the LINC complex protein Nesprin-2G, targeting it for degradation
- BROX coordinates local relaxation of mechanical stress with membrane remodeling

Authors

Samuel S. Wallis,
Leandro N. Ventimiglia,
Evita Otigbah, ..., Sergi Garcia-Manyes,
Juan Martin-Serrano,
Monica Agromayor

Correspondence

juan.martin_serrano@kcl.ac.uk (J.M.-S.),
monica.agromayor@kcl.ac.uk (M.A.)

In brief

Nuclear envelope (NE) integrity is essential to protect the genome from damage. Wallis et al. investigate how the NE is repaired in case of rupture. The study demonstrates that BROX is locally recruited by ESCRTs and reduces cytoskeletal stress through its action on Nesprin-2G to help reseal the ruptured membrane

Wallis et al., 2021, Developmental Cell *56*, 3192–3202
December 6, 2021 © 2021 The Author(s). Published by Elsevier Inc.
https://doi.org/10.1016/j.devcel.2021.10.022

CellPress
OPEN ACCESS

Developmental Cell

Short Article

The ESCRT machinery counteracts Nesprin-2G-mediated mechanical forces during nuclear envelope repair

Samuel S. Wallis,[1,7] Leandro N. Ventimiglia,[1,7] Evita Otigbah,[1,7] Elvira Infante,[2] Miguel Angel Cuesta-Geijo,[1,3] Gururaj Rao Kidiyoor,[4] M. Alejandra Carbajal,[5] Roland A. Fleck,[5] Marco Foiani,[4] Sergi Garcia-Manyes,[2,6] Juan Martin-Serrano,[1,*] and Monica Agromayor[1,8,*]

[1]Department of Infectious Diseases, King's College London, Faculty of Life Sciences & Medicine, London SE1 9RT, UK
[2]Department of Physics, Randall Centre for Cell and Molecular Biophysics, and London Centre for Nanotechnology, King's College London, London WC2R 2LS, UK
[3]Centro Nacional Instituto de Investigación y Tecnología Agraria y Alimentaria (CSIC), Ctra. de la Coruña Km 7.5, 28040 Madrid, Spain
[4]Fondazione Istituto FIRC di Oncologia Molecolare (IFOM), Via Adamello 16, 20139 Milan, Italy; Università degli Studi di Milano, 20122 Milan, Italy
[5]Centre for Ultrastructural Imaging, King's College London, London SE1 1UL, UK
[6]the Francis Crick Institute, 1 Midland Road, London NW1 1AT, UK
[7]These authors contributed equally
[8]Lead contact
*Correspondence: juan.martin_serrano@kcl.ac.uk (J.M.-S.), monica.agromayor@kcl.ac.uk (M.A.)
https://doi.org/10.1016/j.devcel.2021.10.022

SUMMARY

Transient nuclear envelope ruptures during interphase (NERDI) occur due to cytoskeletal compressive forces at sites of weakened lamina, and delayed NERDI repair results in genomic instability. Nuclear envelope (NE) sealing is completed by endosomal sorting complex required for transport (ESCRT) machinery. A key unanswered question is how local compressive forces are counteracted to allow efficient membrane resealing. Here, we identify the ESCRT-associated protein BROX as a crucial factor required to accelerate repair of the NE. Critically, BROX binds Nesprin-2G, a component of the linker of nucleoskeleton and cytoskeleton complex (LINC). This interaction promotes Nesprin-2G ubiquitination and facilitates the relaxation of mechanical stress imposed by compressive actin fibers at the rupture site. Thus, BROX rebalances excessive cytoskeletal forces in cells experiencing NE instability to promote effective NERDI repair. Our results demonstrate that BROX coordinates mechanoregulation with membrane remodeling to ensure the maintenance of nuclear-cytoplasmic compartmentalization and genomic stability.

INTRODUCTION

The nuclear compartment is highly dynamic, and its integrity is constantly challenged by mechanical forces. The response to these forces is modulated by nuclear envelope (NE)-associated proteins including the linker of nucleoskeleton and cytoskeleton complex (LINC), which transmits forces between the cytoskeleton and the nucleus (Lee and Burke, 2018). Besides compression and deformation, the NE undergoes several remodeling events, most notably its complete disassembly and reassembly during mitosis (Güttinger et al., 2009). Transient NE ruptures during interphase (NERDI) also occur upon disruption of the NE organization and mechanical stresses generated by cytoskeletal forces (De Vos et al., 2011; Hatch and Hetzer, 2016; Takaki et al., 2017; Vargas et al., 2012), and failure to repair NERDIs contributes to genomic instability (Cho et al., 2019; Denais et al., 2016; Earle et al., 2020; Irianto et al., 2017; Raab et al., 2016; Xia et al., 2018). Therefore, a tight coordination between membrane remodeling and mechanical forces is critical to preserve NE integrity and protect the genome from damage.

NE ruptures expose the chromatin-associated protein barrier to autointegration factor (BAF) and inner nuclear membrane proteins such as LEMD2, which recruits endosomal sorting complex required for transport (ESCRT) machinery to remodel the damaged membranes (Halfmann et al., 2019; Penfield et al., 2018; Thaller et al., 2019; Young et al., 2020). NERDI repair is completed by local polymerization of ESCRT-III filaments and subsequent constriction by the AAA-ATPase VPS4 seals the NE gap (Denais et al., 2016; Raab et al., 2016). Despite these advances in our understanding of the mechanisms underpinning NE repair, it remains unknown how local regulation of compressive forces at the NE is coordinated with membrane remodeling to re-establish nuclear compartmentalization.

To address this question, we investigated the function of BROX, a conserved Bro1 domain protein that binds ESCRT-III (Ichioka et al., 2008). Here, we identify BROX as a NE-associated

 © 2021 The Author(s). Published by Elsevier Inc.
This is an open access article under the CC BY license (http://creativecommons.org/licenses/by/4.0/).

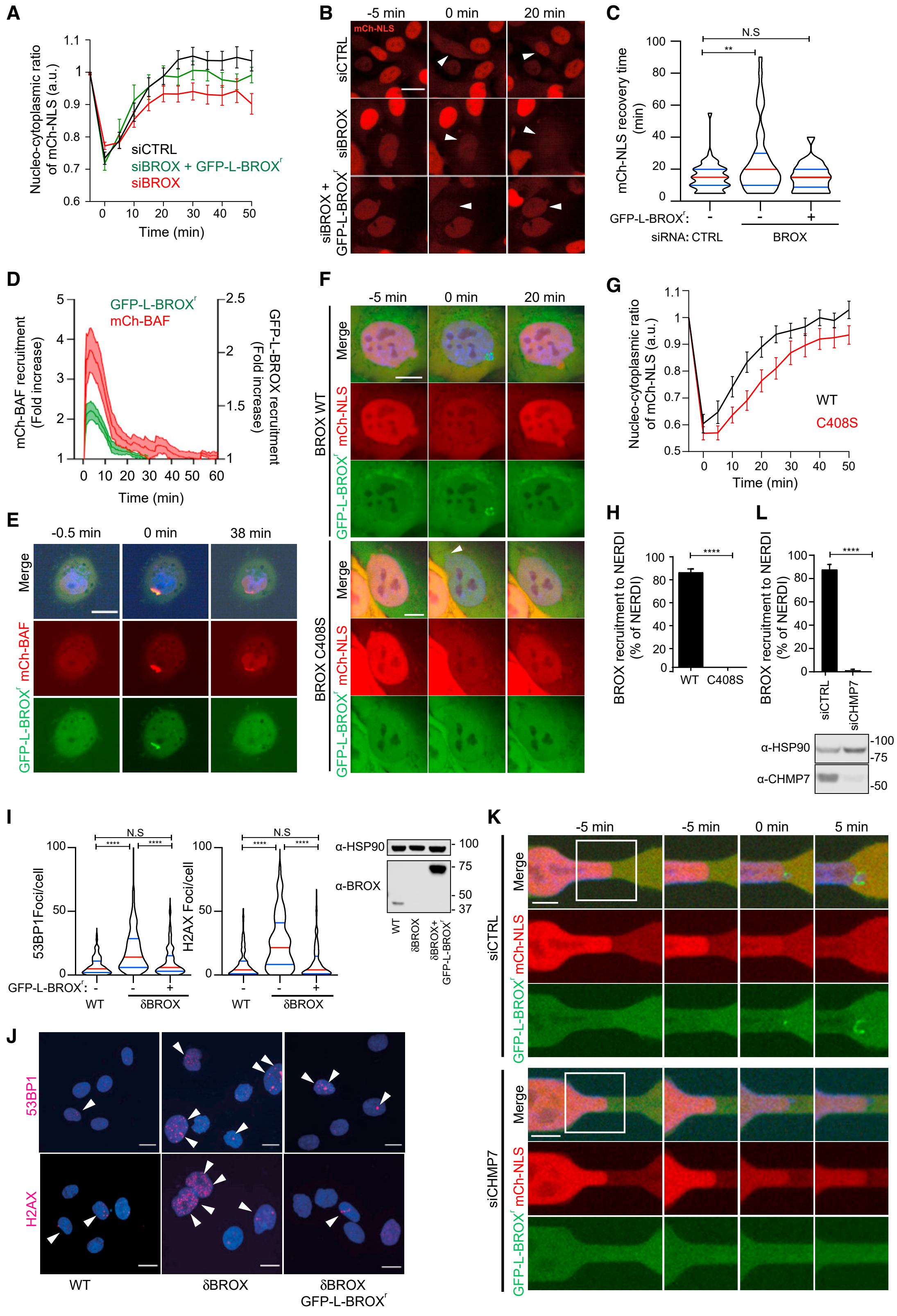

Figure 1. BROX is required for efficient NERDI repair

(A) Recovery of NE integrity after NERDI in cells expressing mCherry-NLS alone or with GFP-L-BROX[r] and treated with control (siCTRL) or BROX-specific siRNA. siCTRL, n = 61; siBROX, n = 60; siBROX + GFP-L-BROX[r], n = 56; $p = 0.0225$ (siCTRL-siBROX), $p \geq 0.05$ (siCTRL-siBROX + WT).

(legend continued on next page)

factor that modulates the biomechanical properties of the nuclear compartment. We demonstrate that BROX counteracts compressive cytoskeletal forces at the NE through the interaction with Nesprin-2G, thus coordinating membrane remodeling and modulation of mechanical forces to facilitate efficient NERDI repair.

RESULTS

BROX is recruited to NERDI events and is required for efficient repair

To directly assess the integrity of the nuclear compartment in cells lacking BROX, HT1080 cells stably expressing mCherry fused to a nuclear localization signal (mCherry-NLS) were treated with BROX-specific siRNA oligos and monitored using time-lapse microscopy. In this system, cytoplasmic accumulation of mCherry-NLS indicates NE rupture, and the time to restore the basal nucleo-cytoplasmic ratio of mCherry-NLS correlates with repair time (Figures 1A and 1B; Video S1). BROX depletion significantly delayed mCherry-NLS nuclear re-accumulation after NERDI as compared with control, while stable expression of siRNA-resistant BROX (GFP-L-$BROX^r$) restored repair times to control levels (Figures 1A–1C and S1A; Video S1). Importantly, GFP-L-$BROX^r$ transiently accumulated at rupture sites, as denoted by DNA herniations and co-localization with mCherry-BAF foci (Denais et al., 2016; Halfmann et al., 2019; Young et al., 2020), supporting a direct role of BROX in NE repair (Figures 1D–1F; Video S2). Mutation of BROX farnesylation site (GFP-L-$BROX^r$C408S) (Ichioka et al., 2008) disrupted BROX association with the NE (Figures S1B and S1C), abolished its recruitment to rupture sites and repair function (Figures 1F–1H, S1D, and S1E; Video S2). Thus, BROX localization to the ruptured membrane is required for efficient NE repair. These observations were further confirmed by deleting the BROX locus using CRISPR-Cas9, as the resulting clone ($HT1080^{\delta BROX}$C1, from here on referred as $HT1080^{\delta BROX}$) showed impaired NERDI repair (Figure S1F; Video S1). $HT1080^{\delta BROX}$ cells also exhibited increased levels of 53BP1 and γ-H2AX foci (Figures 1I and 1J), and similar DNA damage was observed on a second clone ($HT1080^{\delta BROX}$C2) (Figures S1G and S1H), strengthening previous observations that NE repair defects increase DNA damage (Cho et al., 2019; Denais et al., 2016; Irianto et al., 2017; Raab et al., 2016; Xia et al., 2018). This phenotype could be rescued by re-expression of GFP-L-$BROX^r$ in $HT1080^{\delta BROX}$ (Figures 1I and 1J).

We then explored the role of upstream regulators of BROX function. CHMP7 triggers the assembly of ESCRT-III subunits such as CHMP4B at NE fenestrations (Vietri et al., 2015; Olmos et al., 2016). While CHMP7 depletion prevented enrichment of GFP-L-$BROX^r$ at rupture sites (Figures 1K and 1L; Video S3), BROX depletion had no effect on CHMP4B-L-GFP recruitment (Figures S1I and S1J; Video S3), demonstrating that BROX is recruited to the damaged NE by the CHMP7/ESCRT-III axis.

BROX regulates NE morphology and compressive actin cytoskeletal forces at the NE

To better understand the role of BROX in NERDI repair, steady-state changes in the nuclei of cells lacking BROX were studied. BROX depletion increased the proportion of cells showing compressive nuclear actin cables (Figures 2A, 2B, S2A, and S2B) and NE invaginations (Figures S2E–S2H). 3D reconstructions of cells stably expressing GFP-Emerin (Figure 2C) and transmission electron microscopy (TEM) of $HT1080^{\delta BROX}$ cells (Figure 2D) revealed a marked increase of intranuclear tube-like structures (INTs), areas of extreme bending of the NE that were reminiscent of the poorly understood nucleoplasmic reticulum (Drozdz and Vaux, 2017). These phenotypes could be reverted to wild-type conditions by treatment with drugs that inhibit actomyosin contractility (Figures S2C and S2D) or re-expression of GFP-L-$BROX^r$ (Figures S2E–S2H).

These profound changes in nuclear morphology and cytoskeletal organization suggest that BROX might regulate the compressive forces acting on the NE and impact its biomechanical properties. Accordingly, we observed that $HT1080^{\delta BROX}$ nuclei were more prone to rupture than were wild type when constant forces were applied with an AFM tip on the plasma membrane on top of the cell nucleus (Figure 2H). Subsequent constant velocity experiments revealed that the forces required to break through the NE were significantly lower in $HT1080^{\delta BROX}$ cells compared with wild-type cells (Figures 2I and S2I–S2K). In agreement with previous studies (Xia et al., 2018), this difference was phenocopied by cells lacking LaminA/C ($HT1080^{\delta LMNA}$), a key structural component of the nuclear lamina (de Leeuw et al., 2018; Robijns et al., 2016) (Figure 2I). As a critical control, no differences were observed between HT1080 and $HT1080^{\delta BROX}$cells in cellular regions where only the plasma membrane was indented (Figure 2I). Taken together, these observations suggest that the imbalance of cytoskeletal forces at the NE in cells lacking BROX may render nuclei more prone to rupture. Consequently, the frequency of NERDI events was increased in BROX-depleted cells, and this NE instability was reduced to control levels in cells rescued with GFP-L-$BROX^r$ (Figure 2J). Areas of the NE with high Gaussian curvature are

(B) Images of representative rupture events. Arrowheads highlight cells undergoing NERDI. Scale bar, 20 μm.
(C) Quantification of the recovery times for events in (A). **p = 0.0039, $^{N.S}p > 0.05$. Median and quartiles are shown in red and blue, respectively.
(D) Quantification of mCherry-BAF and GFP-L-$BROX^r$WT recruitment to the NE in cells experiencing NERDI (n = 7).
(E and F) Representative images of the quantifications shown in (D) and (G), respectively. Scale bars, 20 μm (E) and 10 μm (F).
(G) Recovery of NE integrity after NERDI in cells expressing mCherry-NLS and GFP-L-$BROX^r$WT (n = 17) or GFP-L-$BROX^r$C408S mutant (n = 15).
(H) Percentage of BROX-decorated NERDIs. WT, n = 71; C408S, n = 49; $p < 0.0001$.
(I) Quantification of 53BP1 and γH2AX-positive foci. n = 147 for each condition; ****$p < 0.0001$, $^{NS}p > 0.05$. Median and quartiles are shown in red and blue, respectively, and cell lysates were examined by blotting with the indicated antibodies.
(J) Representative images of the quantifications shown in (I). Arrowheads show DNA damage foci. Scale bar, 15 μm.
(K) Representative images of the quantifications shown in (L). Scale bar, 5 μm.
(L) Quantification of BROX-decorated NERDIs in siRNA-treated cells co-expressing mCherry-NLS and GFP-L-$BROX^r$ migrating through 4-μm constrictions. Cell lysates were examined by blotting with the indicated antibodies. siCTRL n = 65, siCHMP7 n = 59; $p < 0.0001$.
See also Figure S1

CellPress
OPEN ACCESS

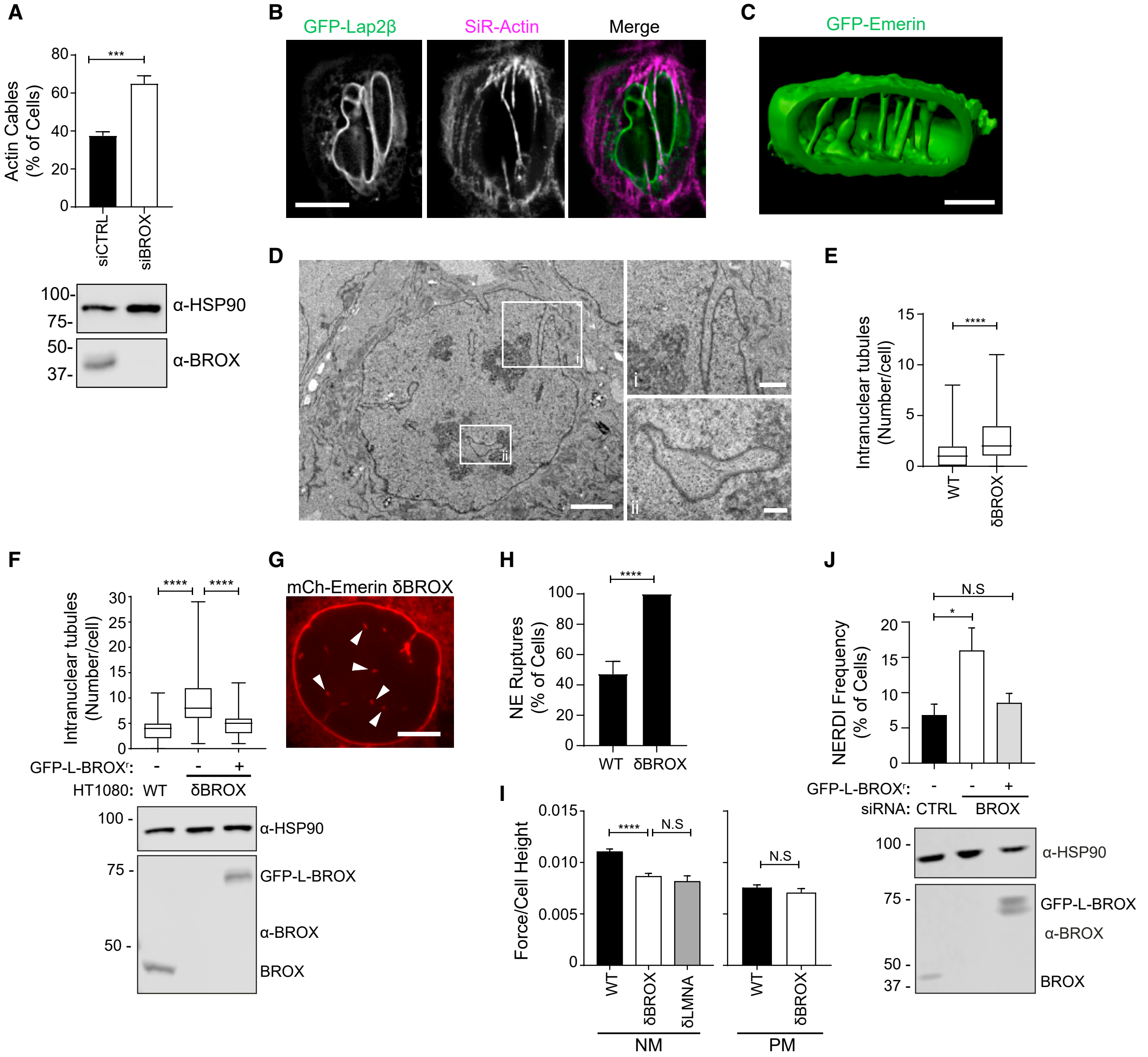

Figure 2. BROX regulates compressive forces at the NE

(A) Quantification of actin cables in HeLa cells expressing GFP-Lap2β treated with the indicated siRNA and stained with SiR-actin. Cell lysates were examined by blotting with the specified antibodies. siCTRL, n = 124; siBROX, n = 104; p = 0.0008.

(B) Representative images of the quantifications shown in (A). Scale bar, 10 μm.

(C) 3D reconstruction of the nucleus of a representative HT1080$^{\delta BROX}$ cell expressing GFP-Emerin. Scale bar, 5 μm.

(D) TEM of a representative HT1080$^{\delta BROX}$ cell. Scale bar, 5 μm. Insets show magnifications of the selected areas. Scale bars, (Di) 1 μm, (Dii) 500 nm.

(E) Quantification of intranuclear tube-like structures (INTs) in wild type (WT; n = 91) or BROX-depleted (δBROX; n = 63) cells imaged by TEM. p < 0.0001.

(F) Quantification of INTs in cells expressing mCherry-Emerin and imaged by confocal microscopy. Rescue experiments were performed by adding GFP-L-BROX^r. WT, n = 157: δBROX, n = 109; δBROX + GFP-L-BROX^r, n = 154; p < 0.0001. Cell lysates were examined by blotting with the indicated antibodies.

(G) Representative image of the quantifications shown in (F). Arrowheads show INTs. Scale bar, 5 μm.

(H) Percentage of NE ruptures in WT (n = 38) and δBROX (n = 22) cells under constant force measured with force-clamp AFM. p ≤ 0.0001.

(I) Quantification of the force required to break through the nuclear (NM, left graph) or plasma membrane (PM, right graph) in HT1080 WT, δBROX, or lamina/C-knockout (δLMNA) cells. WT-NM, n = 141; δBROX-NM, n = 173; δLMNA-NM, n = 58; WT-PM, n = 97; δBROX-PM, n = 94; ****p < 0.0001, $^{N.S}$p > 0.05.

(J) Analysis of NERDI frequency in HT1080 cells expressing mCherry-NLS alone or with GFP-L-BROX^r and treated with the indicated siRNA. Cell lysates were examined by blotting with the specified antibodies. siCTRL, n = 1,019; siBROX, n = 914; siBROX + GFP-L-BROX^r, n = 662; *p = 0.0113, $^{N.S}$p > 0.05.

See also Figure S2.

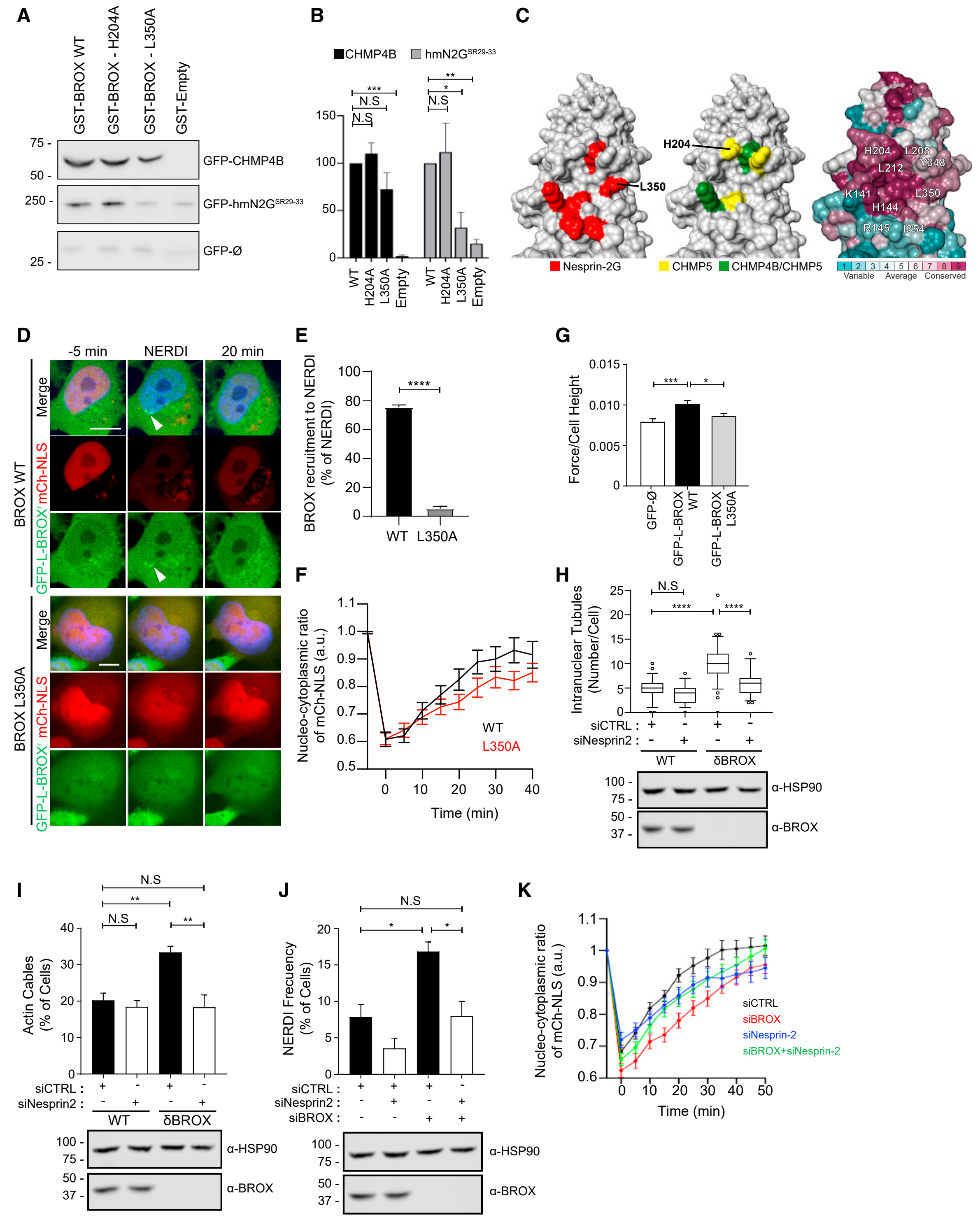

Figure 3. BROX/Nesprin-2G interaction is needed for the release of compressive forces at the NE and rupture repair

(A and B) Pull-down experiments using recombinant GST-tagged wild-type (WT) or mutant (L350A, H204A) BROX as bait and lysates from 293T cells transfected with the indicated GFP constructs as preys. Eluates were analyzed by blotting with α-GFP antibody and band intensity is plotted normalized to BROX WT signal in (B) *p = 0.0192, **p = 0.0036, ***p = 0.001, $^{N.S}$p > 0.05.

(C) Electrostatic surface representations of BROX Bro1 domain showing residues needed for binding to Nesprin-2G (red), CHMP5 (yellow), or both CHMP4B and CHMP5 (green) and conserved residues among BROX proteins in metazoan.

(D) Representative images of the quantifications shown in (E) and (F). Arrowhead highlights BROX recruitment to site of rupture. Scale bar, 10 μm.

(legend continued on next page)

CellPress
OPEN ACCESS

prone to rupture and excess DNA damage (Xia et al., 2018, 2019). Therefore, extreme NE bending may contribute to the NE ruptures in BROX-depleted cells.

The interaction between BROX and Nesprin-2G is required to relax compressive forces at the NE

A genome-wide yeast two-hybrid screen subsequently revealed a potential interaction between BROX and Nesprin-2, a component of the LINC complex (Lee and Burke, 2018). All Nesprin-2 fragments identified contained spectrin repeats 29 to 33(SR29–33), which only appear in giant Nesprin-2(Nesprin-2G) (Rajgor and Shanahan, 2013). BROX interaction with the Nesprin-2G SR29-33 region (Nesprin-2G$^{SR29–33}$) was first confirmed by yeast two-hybrid (Figure S3A). We then engineered human GFP-tagged mini-Nesprin-2G (hmN2G), a functional reporter for Nesprin-2G (Luxton et al., 2010), by adding the SR29–33 region. The resulting construct, GFP-hmN2G$^{SR29–33}$ (Figure S3B), bound GST-BROX in a co-precipitation assay (Figures 3A and 3B), thus confirming the BROX/Nesprin-2G interaction in the NE native environment.

Next, we used existing BROX crystal structures to map the interaction with Nesprin-2G (Mu et al., 2012; Zhai et al., 2011). We performed alanine substitution of exposed residues (Figure 3C) and tested the resulting mutants by yeast two-hybrid (Figure S3A). A BROX residue essential for CHMP5 binding (H204) (Mu et al., 2012) was dispensable for the interaction with Nesprin-2G$^{SR29–33}$ (Figure S3A), thus indicating that CHMP5 and Nesprin-2G bind overlapping but distinct surfaces on BROX, which are evolutionarily conserved. Accordingly, mutation of L350 or I354 rendered BROX unable to bind Nesprin-2G$^{SR29–33}$ without altering its ability to interact with ESCRT-III (Figures 3A and S3A). Co-precipitation experiments confirmed the specific loss of binding between GFP-hmN2G$^{SR29–33}$ and BROX L350A, while GFP-hmN2G$^{SR29–33}$ interaction with BROX H204A was retained (Figures 3A and 3B). Therefore, these studies identified BROX L350A as a specific tool to determine whether loss of Nesprin-2G binding impacts BROX function. Crucially, replacement of endogenous BROX with GFP-L-BROX^{r}L350A abolished BROX recruitment to sites of NE rupture and resulted in impaired NE resealing (Figures 3D–3F, S3C–S3E; Videos S4 and S5), showing that BROX-Nesprin-2G interaction is required for repair. Similarly, the reduced breakthrough forces of the nuclear membrane in HT1080$^{\delta BROX}$GFP-L-BROX^{r}L350A cells recapitulated the phenotype observed in BROX-depleted cells (HT1080$^{\delta BROX}$GFP-ø) (Figure 3G), indicating that BROX-Nesprin-2G interaction regulates the biomechanical properties of the nucleus.

LINC-complex-mediated nuclear compression contributes to NE ruptures (Hatch and Hetzer, 2016). Therefore, we reasoned that BROX may help reduce the mechanical stress on the nucleus by counteracting Nesprin-2G-mediated compressive forces. This model predicts that the increase on compressive forces observed in nuclei of BROX-depleted cells may be reduced upon Nesprin-2G depletion. Consistently, silencing of Nesprin-2G in HT1080$^{\delta BROX}$ cells reverted the increased number of INTs, NE invaginations and nuclear actin cables to wild-type levels (Figures 3H, 3I, and S3F–S3J). As a control for specificity, depletion of Nesprin-2G in wild-type HT1080 cells did not alter the NE compressive forces, as indicated by the number of actin cables observed in this condition (Figure 3I). Importantly, the frequency of NERDI events in BROX-depleted cells was reduced to control levels by co-depleting Nesprin-2G. Moreover, BROX function was dispensable in the context of NE rupture when Nesprin-2G levels were reduced (Figures 3J and 3K; Video S6). Therefore, BROX function in NE homeostasis and repair is largely restricted to the counteraction of compressive forces imposed by Nesprin-2G dysregulation.

Nesprin-2G ubiquitination state and localization at compression sites is mediated by BROX

We observed that GFP-hmN2G$^{SR29–33}$ exhibited increased ubiquitination levels compared with GFP-hmN2G (Figure S4A), suggesting that BROX may counteract excess Nesprin-2G activity by ubiquitination. This model was supported by the specific increase of GFP-hmN2G$^{SR29–33}$ ubiquitination upon FLAG-BROX^{r}WT overexpression, while this effect was not induced by FLAG-BROX^{r}L350A or FLAG-BROX^{r}H204A (Figure 4A). Critically, although GFP-L-BROX^{r}H204A was still recruited to sites of rupture, replacement of endogenous BROX with this mutant resulted in impaired NE repair (Figures 4B, S3E, S4B, and S4C; Video S4), thus providing a strong correlation between BROX NE repair activity and Nesprin-2G ubiquitination.

The p97 AAA + ATPase complex together with the ubiquitin/proteasome system is involved in turnover of NE components (Buchwalter et al., 2019; Tsai et al., 2016). Treatment with the p97 inhibitor EeyarestatinI resulted in the accumulation of ubiquitinated GFP-hmN2G$^{SR29–33}$ (Figure 4C), while inhibition of ER mannosidaseI with kifunensine had no significant effect, suggesting that ubiquitinated Nesprin-2G is targeted for ER-associated degradation (ERAD) without entering the ER lumen (Fagioli

(E) Percentage of BROX-decorated NERDIs in cells co-expressing mCherry-NLS and GFP-tagged wild type (WT; n = 38) or mutant BROX (L350A; n = 48), $p < 0.0001$.

(F) Recovery of NE integrity after NERDI. WT, n = 33; L350A, n = 47; p = 0.0498.

(G) Measurements of the force required to break through the NE in cells expressing GFP-L-BROX^{r} WT (n = 110) or L350A (n = 76) and normalized by the cell height in each case. Cells expressing GFP-ø (n = 75) were used as a control. ***p = 0.0006, *p = 0.0293, $^{N.S}p > 0.05$.

(H) Quantification of INTs in WT or δBROX cells expressing GFP-Emerin after treatment with the indicated siRNAs. WT + siCTRL, n = 69; WT + siNesprin-2, n = 56; δBROX + siCTRL, n = 67; δBROX + siNesprin-2, n = 47. Cell lysates were analyzed by blotting with the indicated antibodies. ****p < 0.0001, $^{N.S}p > 0.05$.

(I) Analysis of nuclear actin cables in WT or δBROX cells treated with the indicated siRNA and stained with SiR-actin. WT + siCTRL, n = 110; WT + siNesprin-2, n = 93, δBROX + siCTRL, n = 83; δBROX + siNesprin-2, n = 68; **p < 0.01, $^{N.S}p > 0.05$. Cell lysates were examined by blotting with the indicated antibodies.

(J) Quantification of percentage of cells showing NERDI. siCTRL, n = 101; siBROX, n = 99; siNesprin-2, n = 59, siBROX + siNesprin-2, n = 66; *p < 0.05, $^{N.S}p > 0.05$. Cell lysates were examined by blotting with the indicated antibodies.

(K) Recovery of NE integrity after NERDI. siCTRL, n = 51; siBROX, n = 56; siNesprin-2, n = 29, siBROX + siNesprin-2, n = 56; p = 0.0027 (siCTRL-siBROX); p < 0.05 (siNesprin-2-siBROX + siNesprin-2).

See also Figure S3.

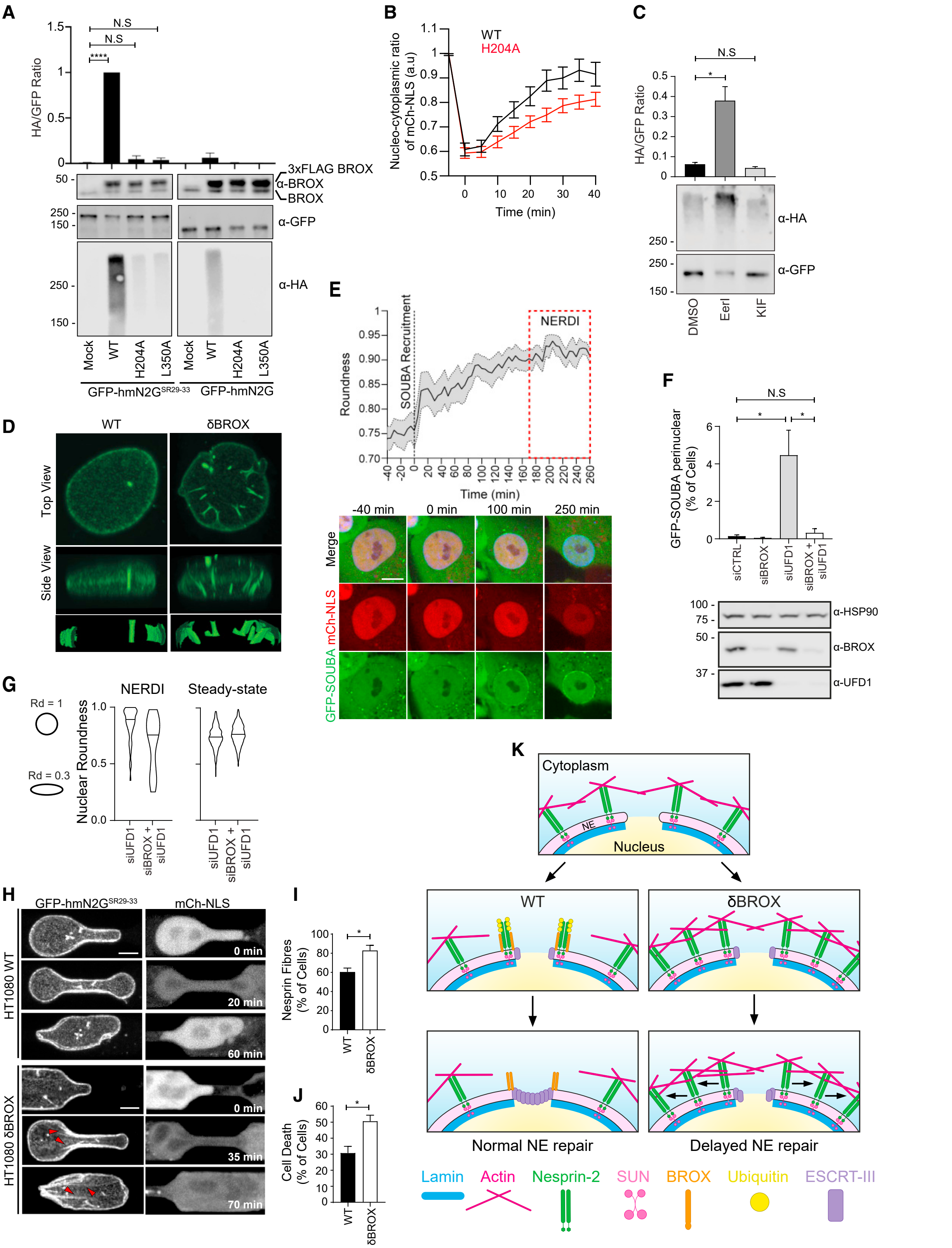

(legend on next page)

and Sitia, 2001; Wang et al., 2008). We then reasoned that BROX may regulate compressive forces at the NE by controlling steady-state levels of Nesprin-2G. In agreement with this model, stably expressed GFP-hmN2G$^{SR29–33}$ was upregulated upon BROX depletion (Figure S4D), and this resulted in the accumulation of INTs decorated with Nesprin-2G (Figures 4D and S4E).

To further test our model, we developed a fluorescent sensor to visualize ubiquitin in live cells by fusing the high-affinity ubiquitin-binding SOUBA domain from UBAP1 (Agromayor et al., 2012) to GFP(GFP-SOUBA) (Figures S4F and S4G). To validate this sensor, ESCRT-mediated degradation of ubiquitinated cargo was inhibited by depleting VPS4, resulting in large cytosolic vesicular compartments decorated by GFP-SOUBA that were reminiscent of enlarged endosomal compartments (Doyotte et al., 2005) (Figure S4G). GFP-SOUBA clusters also appeared in cells treated with siRNA against UFD1, a p97 cofactor needed for ERAD-mediated proteosomal degradation (Meyer et al., 2002) (Figure S4G). We therefore hypothesized that UFD1 depletion could be used to capture transient ubiquitination events during NERDI. Accordingly, a striking perinuclear accumulation of GFP-SOUBA was observed during the time preceding a NERDI event, and this was abrogated in BROX-depleted cells (Figures 4E and 4F; Video S7). Similarly, the perinuclear GFP-SOUBA signal was reduced by Nesprin-2G co-depletion (Figure S4H), supporting BROX-dependent ubiquitination of Nesprin-2G in this context. Furthermore, perinuclear GFP-SOUBA enrichment was restricted to cells experiencing NERDIs (Figure S4I), indicating that steady-state levels of BROX at the NE (Figures S1B and S1C) induce ubiquitination in unstable nuclei, which will later rupture. Importantly, UFD1 depletion did not increase NERDI frequency (Figure S4J). GFP-SOUBA accumulation around the nucleus was associated with progressive rounding of the nucleus before a rupture event (Figure 4E; Video S7), and this change was less marked in BROX-depleted cells (Figure 4G). Increases in nuclear roundness correlate with inhibition of actomyosin contractility (Takaki et al., 2017), thus suggesting that BROX rebalances the forces exerted by the cytoskeleton on the nucleus in cells experiencing NE instability. In further support for this model, BROX-depleted cells migrating through narrow constrictions showed an increased number of GFP-hmN2G$^{SR29–33}$fibers at sites of compression (Figures 4H and 4I; Video S8). These fibers are comparable to the structures observed at the surface of the nucleus in cells that have a GFP-tag added to the endogenous Nesprin-2 gene, which are mechanically linked with actin (Davidson et al., 2020). Moreover, the observed increase in compressive forces correlated with the inability of HT1080$^{\delta BROX}$cells to repair their nuclear ruptures and with higher death frequency (Figure 4J), a phenotype that resembles the migration-induced apoptosis observed in cells with decreased levels of LaminA (Harada et al., 2014).

DISCUSSION

Our study demonstrates that BROX regulates NE homeostasis by restricting the cytoskeletal forces imposed by Nesprin-2G. BROX silencing destabilizes the delicate mechanical balance of the NE, favoring the prevalence of actin-driven compressive forces. This mechanical dysregulation increases the susceptibility of BROX-silenced cells to suffer NERDI and impairs the efficient repair of these ruptures, ultimately compromising genome stability. Intriguingly, haploinsufficiency of BROX is associated with familial non-medullary thyroid cancer (Pasquali et al., 2021); thus, it is conceivable that NE instability associated with BROX deficiencies may contribute to tumor cell invasion (Nader et al., 2021).

We propose a model (Figure 4K) whereby BROX is recruited to the sites of NE rupture by the CHMP7/ESCRT-III membrane repair machinery to trigger the ubiquitination of Nesprin-2G. As consequence, the LINC complex may uncouple from the cytoskeleton at sites of actin-mediated compression during NE resealing, facilitating the final closure of wounded nuclear membranes. In the absence of BROX, dysregulated Nesprin-2G activity may increase local actin-driven compression at the rupture site, generating tensile forces that pull the ruptured membranes apart. Thus, the lack of coordination between membrane

Figure 4. BROX regulates Nesprin-2G protein levels at compression sites

(A) GFP pull-down of cells stably expressing GFP-hmN2G$^{SR29–33}$ or GFP-hmN2G and transfected with HA-ubiquitin and FLAG-tagged BROX either wild-type or mutant (WT, H204A, and L350A) constructs. Eluted fractions were examined by blotting with the indicated antibodies. Samples were adjusted for loading of equal amounts of GFP-hmN2G constructs and ratio between HA and GFP bands intensity was plotted. Mock = non-transfected control. ****$p < 0.0001$, $^{N.S}p > 0.05$.

(B) Recovery of NE integrity after NERDI in cells co-expressing mCherry-NLS and GFP-L-BROX^{r} wild-type (WT; n = 33) or mutant (H204A; n = 59). The movies used to quantify recovery in WT were the same as in Figure 3F. p = 0.0132.

(C) GFP pull-down experiments of 293T transfected with HA-ubiquitin and GFP-hmN2G$^{SR29–33}$ and treated with DMSO, eeyarstatin or kifunensine. Eluted fractions were examined by blotting with the indicated antibodies and ratio between HA and GFP bands intensity was plotted; *p = 0.0039, $^{N.S}p > 0.05$.

(D) Images of representative WT or δBROX cells expressing GFP-hmN2G$^{SR29–33}$. Z series are displayed as volumetric view and 3D reconstruction.

(E) Analysis of nuclear morphology in UFD1-depleted cells co-expressing mCherry-NLS and GFP-SOUBA. Graph shows nuclear roundness over time in cells showing perinuclear GFP-SOUBA accumulation (n = 10). The box denotes the time frame of NERDI events. Representative images from the indicated times are shown. Scale bar, 10 μm.

(F) Percentage of cells with perinuclear GFP-SOUBA accumulation in cells treated with siCTRL (n = 2,016), siBROX (n = 1,929), siUFD1 (n = 1,945) or siBROX + siUFD1 (n = 1,746); *$p < 0.05$, $^{N.S}p > 0.05$. Cell lysates were examined by blotting with the indicated antibodies

(G) Quantification of nuclear roundness (Rd) at the point of NERDI (middle panel; siUFD1, n = 43; siUFD1 + siBROX, n = 16) or at steady state (right panel: siUFD1, n = 643; siUFD1 + siBROX, n = 983). Diagrams show example nucleus shapes and associated roundness.

(H) Representative images of WT or δBROX cells expressing GFP-hmN2G$^{SR29–33}$ and mCherry-NLS migrating through narrow constrictions. Scale bar, 5 μm. Arrowheads indicate GFP-hmN2G$^{SR29–33}$ fibers.

(I and J) Quantification of the percentage of cells with GFP-hmN2G$^{SR29–33}$ fibers (I) and of cells dying (J) as they migrate through constrictions. WT, n = 101; δBROX, n = 86; *$p < 0.05$.

(K) Proposed model.

See also Figure S4.

remodeling and mechanical force regulation delays NERDI repair, exposing the genome to cytoplasmic nucleases that cause genetic instability. Giant Nesprin proteins can function at the NE independently of LINC complexes by maintaining cellular connectivity across the cytoplasm (Hao et al., 2021), a function that may not be fully recapitulated by the mini-nesprin approach used in our study. Also, a clear low GFP-Nesprin signal is visible in the cytoplasm of genetically engineered cells (Davidson et al., 2020), suggesting that BROX may further regulate the overall mechanical integrity of the cell in a LINC-independent manner by stabilizing the distal cytosolic nesprin-based meshwork, which could indirectly function as a buffer against forces exerted on the nucleus.

HT1080$^{\delta BROX}$ cells stably expressing GFP-hmN2G$^{SR29–33}$ show lower post-migration survival than parental cells expressing the same construct when migrating through narrow constrictions, suggesting a protective role of BROX in cells under migration stresses. It has been previously shown that high contractility conditions stabilize LaminA to shield the genome from DNA damage by retaining repair factors in the nucleus (Cho et al., 2019; Xia et al., 2019). Whether similar DNA protection mechanisms are activated in the absence of BROX needs further investigation. Of note, HT1080 cells stably expressing GFP-hmN2G$^{SR29–33}$ show increased cell death when migrating through constricted microchannels as compared with standard 2D culture, indicating that stable overexpression of the mini-Nesprin-2G construct could be altering the cell's mechanical stiffness and susceptibility to stress by perhaps modulating endogenous LINC complexes.

Recent work has proposed that the nucleus functions as a ruler that senses changes in cell shape as consequence of externally applied mechanical stimuli (Lomakin et al., 2020; Venturini et al., 2020). The unfolding of NE invaginations and wrinkles by external forces triggers calcium-dependent activation of cytosolic phospholipase A2 (cPLA2). This mechanism induces cortical myosin II contractility, allowing the cells to rapidly tune their cytoskeletal network in response to changes in environmental conditions. The aberrant nuclear morphology in BROX-silenced cells suggests that the BROX-Nesprin-2G interaction could play a major role in mechanosensing by regulating nuclear shape, perhaps modulating the calcium-dependent activation of cPLA2 and/or the induction of cortical myosin II contractility.

Limitations of the study

We have depleted BROX from cells using two complementary approaches, CRISPR and siRNA. Of the two knockout clones selected for our studies, HT1080$^{\delta BROX}$C1 has a higher DNA content than the parental HT1080 cell line, while HT1080$^{\delta BROX}$C2 has the same DNA content. Both clones show similar defects in NE morphology and DNA damage that can be rescued by re-expressing BROX and recapitulate the phenotypes observed upon siRNA-mediated BROX depletion, showing that DNA content does not influence the magnitude of the phenotypes observed. However, although the reduced breakthrough forces of the nuclear membrane observed in HT1080$^{\delta BROX}$C1 cells can be rescued by re-expressing BROX, we cannot fully exclude that DNA content influences the nuclear mechanics probed by the AFM tip, as this has only been tested with HT1080$^{\delta BROX}$C1 cells.

STAR★METHODS

Detailed methods are provided in the online version of this paper and include the following:

- KEY RESOURCES TABLE
- RESOURCE AVAILABILITY
  - Lead contact
  - Materials availability
  - Data and code availability
- EXPERIMENTAL MODEL AND SUBJECT DETAILS
  - Cell lines
- METHOD DETAILS
  - Plasmids
  - Generation of stable cell lines
  - Generation of HT1080$^{\delta BROX}$ cell line
  - Transfections
  - Western blotting
  - qPCR
  - Yeast-two hybrid assays
  - Analysis of BROX conservation and 3D modelling
  - Mechanical perturbation of cells using an AFM
  - Breakthrough forces experiments
  - Coprecipitation assays
  - Flow cytometry
  - Transmission Electron Microscopy (TEM)
  - Immunofluorescence
- QUANTIFICATION AND STATISTICAL ANALYSIS
  - Image analysis
  - NERDI analysis in unconstrained migration
  - NERDI analysis using confinement microchannels
  - Quantification of 53BP1 and γH2AX foci
  - Quantification of actin cables
  - Quantification of NE invaginations and intranuclear tubules (INTs)
  - Quantification of fluorescence intensity profiles
  - Analysis of perinuclear GFP-SOUBA accumulation and nuclear rounding quantification
  - Quantification of hmN2G$^{SR29-33}$ cables and cell death
  - Statistical analysis

SUPPLEMENTAL INFORMATION

Supplemental information can be found online at https://doi.org/10.1016/j.devcel.2021.10.022.

ACKNOWLEDGMENTS

The authors thank W. H. DeVos for kindly providing the lamina/C knockout cells, the UK NIHR Comprehensive BRC at KCL for an equipment grant, and the Nikon Imaging Centre at KCL for technical support in imaging. This work was supported by awards from the BBSRC (BB/N000501/1) and the King's Health Partners R&D research fund (R151001) to M.A.; the Wellcome Trust (WT102871MA) to J.M.-S.; the European Commission (FET Proactive 731957), EPSRC (EP/M022536/1), Leverhulme Trust (RPG-2015-225 and RL-2016-015), Wellcome Trust (212218/Z/18/Z) and Royal Society (RSWF/R3/183006) to S.G.-M.; and the Italian Association for Cancer Research (AIRC) to M.F. S.S.W. was supported by the King's Bioscience Institute and the Guy's and St Thomas' Charity Prize Ph.D. Programme in Biomedical and Translational Science. E.O. is supported by the UK Medical Research Council (MR/N013700/1) and is a King's College London member of the MRC Doctoral Training Partnership in Biomedical Sciences.

AUTHOR CONTRIBUTIONS

S.S.W., L.N.V., E.O., J.M.-S., and M.A. conceived the study. E.I. and S.G.-M. designed, performed, and analyzed AFM experiments. M.A.C. and R.A.F. designed, performed, and analyzed electron microscopy experiments. S.S.W., L.N.V., E.O., and M.A.C.-G. generated tools and performed and analyzed data from all other experiments. G.R.K. and M.F. helped with the design and analysis of experiments using microchannels. M.A. and J.M.-S. analyzed data and wrote the manuscript with assistance from all other authors.

DECLARATION OF INTERESTS

The authors declare no competing interests.

Received: July 14, 2020
Revised: August 3, 2021
Accepted: October 28, 2021
Published: November 23, 2021

REFERENCES

Agromayor, M., Carlton, J.G., Phelan, J.P., Matthews, D.R., Carlin, L.M., Ameer-Beg, S., Bowers, K., and Martin-Serrano, J. (2009). Essential role of hIST1 in cytokinesis. Mol. Biol. Cell *20*, 1374–1387.

Agromayor, M., and Martin-Serrano, J. (2006). Interaction of AMSH with ESCRT-III and deubiquitination of endosomal cargo. J. Biol. Chem. *281*, 23083–23091.

Agromayor, M., Soler, N., Caballe, A., Kueck, T., Freund, S.M., Allen, M.D., Bycroft, M., Perisic, O., Ye, Y., McDonald, B., et al. (2012). The UBAP1 subunit of ESCRT-I interacts with ubiquitin via a SOUBA domain. Structure *20*, 414–428.

Ashkenazy, H., Abadi, S., Martz, E., Chay, O., Mayrose, I., Pupko, T., and Ben-Tal, N. (2016). ConSurf 2016: an improved methodology to estimate and visualize evolutionary conservation in macromolecules. Nucleic Acids Res *44*, W344–W350.

Atilla-Gokcumen, G.E., Muro, E., Relat-Goberna, J., Sasse, S., Bedigian, A., Coughlin, M.L., Garcia-Manyes, S., and Eggert, U.S. (2014). Dividing cells regulate their lipid composition and localization. Cell *156*, 428–439.

Bogerd, H.P., Fridell, R.A., Blair, W.S., and Cullen, B.R. (1993). Genetic evidence that the Tat proteins of human immunodeficiency virus types 1 and 2 can multimerize in the eukaryotic cell nucleus. J. Virol. *67*, 5030–5034.

Buchwalter, A., Schulte, R., Tsai, H., Capitanio, J., and Hetzer, M. (2019). Selective clearance of the inner nuclear membrane protein emerin by vesicular transport during ER stress. eLife *8*, e49796.

Carlton, J.G., Caballe, A., Agromayor, M., Kloc, M., and Martin-Serrano, J. (2012). ESCRT-III governs the Aurora B-mediated abscission checkpoint through CHMP4C. Science *336*, 220–225.

Cho, S., Vashisth, M., Abbas, A., Majkut, S., Vogel, K., Xia, Y., Ivanovska, I.L., Irianto, J., Tewari, M., Zhu, K., et al. (2019). Mechanosensing by the lamina protects against nuclear rupture, DNA damage, and cell-cycle arrest. Dev. Cell *49*, 920–935.e5.

Davidson, P.M., Battistella, A., Déjardin, T., Betz, T., Plastino, J., Borghi, N., Cadot, B., and Sykes, C. (2020). Nesprin-2 accumulates at the front of the nucleus during confined cell migration. EMBO Rep *21*, e49910.

de Leeuw, R., Gruenbaum, Y., and Medalia, O. (2018). Nuclear lamins: thin filaments with major functions. Trends Cell Biol *28*, 34–45.

De Vos, W.H., Houben, F., Kamps, M., Malhas, A., Verheyen, F., Cox, J., Manders, E.M., Verstraeten, V.L., van Steensel, M.A., Marcelis, C.L., et al. (2011). Repetitive disruptions of the nuclear envelope invoke temporary loss of cellular compartmentalization in laminopathies. Hum. Mol. Genet. *20*, 4175–4186.

Denais, C.M., Gilbert, R.M., Isermann, P., McGregor, A.L., te Lindert, M., Weigelin, B., Davidson, P.M., Friedl, P., Wolf, K., and Lammerding, J. (2016). Nuclear envelope rupture and repair during cancer cell migration. Science *352*, 353–358.

Doyotte, A., Russell, M.R., Hopkins, C.R., and Woodman, P.G. (2005). Depletion of TSG101 forms a mammalian "Class E" compartment: a multicisternal early endosome with multiple sorting defects. J. Cell Sci. *118*, 3003–3017.

Drozdz, M.M., and Vaux, D.J. (2017). Shared mechanisms in physiological and pathological nucleoplasmic reticulum formation. Nucleus *8*, 34–45.

Earle, A.J., Kirby, T.J., Fedorchak, G.R., Isermann, P., Patel, J., Iruvanti, S., Moore, S.A., Bonne, G., Wallrath, L.L., and Lammerding, J. (2020). Mutant lamins cause nuclear envelope rupture and DNA damage in skeletal muscle cells. Nat. Mater. *19*, 464–473.

Fagioli, C., and Sitia, R. (2001). Glycoprotein quality control in the endoplasmic reticulum. Mannose trimming by endoplasmic reticulum mannosidase I times the proteasomal degradation of unassembled immunoglobulin subunits. J. Biol. Chem. *276*, 12885–12892.

Güttinger, S., Laurell, E., and Kutay, U. (2009). Orchestrating nuclear envelope disassembly and reassembly during mitosis. Nat. Rev. Mol. Cell Biol. *10*, 178–191.

Halfmann, C.T., Sears, R.M., Katiyar, A., Busselman, B.W., Aman, L.K., Zhang, Q., O'Bryan, C.S., Angelini, T.E., Lele, T.P., and Roux, K.J. (2019). Repair of nuclear ruptures requires barrier-to-autointegration factor. J. Cell Biol. *218*, 2136–2149.

Hao, H., Kalra, S., Jameson, L.E., Guerrero, L.A., Cain, N.E., Bolivar, J., and Starr, D.A. (2021). The Nesprin-1/-2 ortholog ANC-1 regulates organelle positioning in C. elegans independently from its KASH or actin-binding domains. eLife *10*, e61069.

Harada, T., Swift, J., Irianto, J., Shin, J.W., Spinler, K.R., Athirasala, A., Diegmiller, R., Dingal, P.C., Ivanovska, I.L., and Discher, D.E. (2014). Nuclear lamin stiffness is a barrier to 3D migration, but softness can limit survival. J. Cell Biol. *204*, 669–682.

Harper, J.W., Adami, G.R., Wei, N., Keyomarsi, K., and Elledge, S.J. (1993). The p21 Cdk-interacting protein Cip1 is a potent inhibitor of G1 cyclin-dependent kinases. Cell *75*, 805–816.

Hatch, E.M., and Hetzer, M.W. (2016). Nuclear envelope rupture is induced by actin-based nucleus confinement. J. Cell Biol. *215*, 27–36.

Ichioka, F., Kobayashi, R., Katoh, K., Shibata, H., and Maki, M. (2008). Brox, a novel farnesylated Bro1 domain-containing protein that associates with charged multivesicular body protein 4 (CHMP4). FEBS Journal *275*, 682–692.

Irianto, J., Xia, Y., Pfeifer, C.R., Athirasala, A., Ji, J., Alvey, C., Tewari, M., Bennett, R.R., Harding, S.M., Liu, A.J., et al. (2017). DNA damage follows repair factor depletion and portends genome variation in cancer cells after pore migration. Curr. Biol. *27*, 210–223.

Jayo, A., Malboubi, M., Antoku, S., Chang, W., Ortiz-Zapater, E., Groen, C., Pfisterer, K., Tootle, T., Charras, G., Gundersen, G.G., et al. (2016). Fascin regulates nuclear movement and deformation in migrating cells. Dev. Cell *38*, 371–383.

Lee, Y.L., and Burke, B. (2018). LINC complexes and nuclear positioning. Semin. Cell Dev. Biol. *82*, 67–76.

Lomakin, A.J., Cattin, C.J., Cuvelier, D., Alraies, Z., Molina, M., Nader, G.P.F., Srivastava, N., Sáez, P.J., Garcia-Arcos, J.M., Zhitnyak, I.Y., et al. (2020). The nucleus acts as a ruler tailoring cell responses to spatial constraints. Science *370*, eaba2894.

Luxton, G.W., Gomes, E.R., Folker, E.S., Vintinner, E., and Gundersen, G.G. (2010). Linear arrays of nuclear envelope proteins harness retrograde actin flow for nuclear movement. Science *329*, 956–959.

Maciejowski, J., Li, Y., Bosco, N., Campbell, P.J., and de Lange, T. (2015). Chromothripsis and kataegis induced by telomere crisis. Cell *163*, 1641–1654.

Martin-Serrano, J., Yarovoy, A., Perez-Caballero, D., and Bieniasz, P.D. (2003). Divergent retroviral late-budding domains recruit vacuolar protein sorting factors by using alternative adaptor proteins. Proc. Natl. Acad. Sci. USA *100*, 12414–12419.

Meyer, H.H., Wang, Y., and Warren, G. (2002). Direct binding of ubiquitin conjugates by the mammalian p97 adaptor complexes, p47 and Ufd1-Npl4. EMBO J *21*, 5645–5652.

Mu, R., Dussupt, V., Jiang, J., Sette, P., Rudd, V., Chuenchor, W., Bello, N.F., Bouamr, F., and Xiao, T.S. (2012). Two distinct binding modes define the interaction of Brox with the C-terminal tails of CHMP5 and CHMP4B. Structure *20*, 887–898.

Nader, G.P.F., Agüera-Gonzalez, S., Routet, F., Gratia, M., Maurin, M., Cancila, V., Cadart, C., Palamidessi, A., Ramos, R.N., San Roman, M., et al. (2021). Compromised nuclear envelope integrity drives TREX1-dependent DNA damage and tumor cell invasion. Cell *184*, 5230–5246.e22. https://doi.org/10.1016/j.cell.2021.08.035.

Olmos, Y., Hodgson, L., Mantell, J., Verkade, P., and Carlton, J.G. (2015). ESCRT-III controls nuclear envelope reformation. Nature *522*, 236–239.

Olmos, Y., Perdrix-Rosell, A., and Carlton, J.G. (2016). Membrane binding by CHMP7 coordinates ESCRT-III-dependent nuclear envelope reformation. Curr. Biol. *26*, 2635–2641.

Pasquali, D., Torella, A., Accardo, G., Esposito, D., Del Vecchio Blanco, F., Salvatore, D., Sabatino, P., Pacini, F., Barbato, F., Castagna, M.G., et al. (2021). BROX haploinsufficiency in familial nonmedullary thyroid cancer. J. Endocrinol. Invest. *44*, 165–171.

Penfield, L., Wysolmerski, B., Mauro, M., Farhadifar, R., Martinez, M.A., Biggs, R., Wu, H.Y., Broberg, C., Needleman, D., and Bahmanyar, S. (2018). Dynein pulling forces counteract lamin-mediated nuclear stability during nuclear envelope repair. Mol. Biol. Cell *29*, 852–868.

Pettersen, E.F., Goddard, T.D., Huang, C.C., Couch, G.S., Greenblatt, D.M., Meng, E.C., and Ferrin, T.E. (2004). UCSF Chimera–a visualization system for exploratory research and analysis. J. Comput. Chem. *25*, 1605–1612.

Raab, M., Gentili, M., de Belly, H., Thiam, H.R., Vargas, P., Jimenez, A.J., Lautenschlaeger, F., Voituriez, R., Lennon-Duménil, A.M., Manel, N., and Piel, M. (2016). ESCRT III repairs nuclear envelope ruptures during cell migration to limit DNA damage and cell death. Science *352*, 359–362.

Rajgor, D., and Shanahan, C.M. (2013). Nesprins: from the nuclear envelope and beyond. Expert Rev. Mol. Med. *15*, e5.

Robijns, J., Molenberghs, F., Sieprath, T., Corne, T.D., Verschuuren, M., and De Vos, W.H. (2016). In silico synchronization reveals regulators of nuclear ruptures in lamin A/C deficient model cells. Sci. Rep. *6*, 30325.

Sanner, M.F., Olson, A.J., and Spehner, J.C. (1996). Reduced surface: an efficient way to compute molecular surfaces. Biopolymers *38*, 305–320.

Schindelin, J., Arganda-Carreras, I., Frise, E., Kaynig, V., Longair, M., Pietzsch, T., Preibisch, S., Rueden, C., Saalfeld, S., Schmid, B., et al. (2012). Fiji: an open-source platform for biological-image analysis. Nat. Methods *9*, 676–682.

Takaki, T., Montagner, M., Serres, M.P., Le Berre, M., Russell, M., Collinson, L., Szuhai, K., Howell, M., Boulton, S.J., Sahai, E., and Petronczki, M. (2017). Actomyosin drives cancer cell nuclear dysmorphia and threatens genome stability. Nat. Commun. *8*, 16013.

Thaller, D.J., Allegretti, M., Borah, S., Ronchi, P., Beck, M., and Lusk, C.P. (2019). An ESCRT-LEM protein surveillance system is poised to directly monitor the nuclear envelope and nuclear transport system. eLife *8*, e45284.

Treier, M., Staszewski, L.M., and Bohmann, D. (1994). Ubiquitin-dependent c-Jun degradation in vivo is mediated by the delta domain. Cell *78*, 787–798.

Tsai, P.L., Zhao, C., Turner, E., and Schlieker, C. (2016). The lamin B receptor is essential for cholesterol synthesis and perturbed by disease-causing mutations. eLife *5*, e16011.

Vargas, J.D., Hatch, E.M., Anderson, D.J., and Hetzer, M.W. (2012). Transient nuclear envelope rupturing during interphase in human cancer cells. Nucleus *3*, 88–100.

Ventimiglia, L.N., Cuesta-Geijo, M.A., Martinelli, N., Caballe, A., Macheboeuf, P., Miguet, N., Parnham, I.M., Olmos, Y., Carlton, J.G., Weissenhorn, W., and Martin-Serrano, J. (2018). CC2D1B coordinates ESCRT-III activity during the mitotic reformation of the nuclear envelope. Dev. Cell *47*, 547–563.e6.

Venturini, V., Pezzano, F., Català Castro, F., Häkkinen, H.-M., Jiménez-Delgado, S., Colomer-Rosell, M., Marro, M., Tolosa-Ramon, Q., Paz-López, S., Valverde, M.A., et al. (2020). The nucleus measures shape changes for cellular proprioception to control dynamic cell behavior. Science *370*.

Vietri, M., Schink, K.O., Campsteijn, C., Wegner, C.S., Schultz, S.W., Christ, L., Thoresen, S.B., Brech, A., Raiborg, C., and Stenmark, H. (2015). Spastin and ESCRT-III coordinate mitotic spindle disassembly and nuclear envelope sealing. Nature *522*, 231–235.

Wang, Q., Li, L., and Ye, Y. (2008). Inhibition of p97-dependent protein degradation by Eeyarestatin I. J. Biol. Chem. *283*, 7445–7454.

Xia, Y., Ivanovska, I.L., Zhu, K., Smith, L., Irianto, J., Pfeifer, C.R., Alvey, C.M., Ji, J., Liu, D., Cho, S., et al. (2018). Nuclear rupture at sites of high curvature compromises retention of DNA repair factors. J. Cell Biol. *217*, 3796–3808.

Xia, Y., Pfeifer, C.R., Zhu, K., Irianto, J., Liu, D., Pannell, K., Chen, E.J., Dooling, L.J., Tobin, M.P., Wang, M., et al. (2019). Rescue of DNA damage after constricted migration reveals a mechano-regulated threshold for cell cycle. J. Cell Biol. *218*, 2545–2563.

Yokokawa, M., Takeyasu, K., and Yoshimura, S.H. (2008). Mechanical properties of plasma membrane and nuclear envelope measured by scanning probe microscope. J. Microsc. *232*, 82–90.

Young, A.M., Gunn, A.L., and Hatch, E.M. (2020). BAF facilitates interphase nuclear membrane repair through recruitment of nuclear transmembrane proteins. Mol. Biol. Cell *31*, 1551–1560.

Zhai, Q., Landesman, M.B., Robinson, H., Sundquist, W.I., and Hill, C.P. (2011). Structure of the Bro1 domain protein BROX and functional analyses of the ALIX Bro1 domain in HIV-1 budding. PLoS One *6*, e27466.

CellPress
OPEN ACCESS

STAR★METHODS

KEY RESOURCES TABLE

REAGENT or RESOURCE	SOURCE	IDENTIFIER
**Antibodies**		
Rabbit polyclonal anti-BROX	Atlas Antibodies	Cat# HPA031445; RRID: AB_10602697
Mouse monoclonal anti-HSP90α/β	Santa Cruz Biotechnology	Cat# sc-13119; RRID: AB_675659)
Rabbit polyclonal anti-CHMP7	Proteintech	Cat# 16424-1-AP; RRID: AB_2079500
Mouse monoclonal anti-GFP (clones 7.1 and 13.1)	Roche	Cat# 11814460001; RRID: AB_390913
Rabbit polyclonal anti-HA	Antibodies Online	Cat# ABIN100176; RRID: AB_10779560
Rabbit polyclonal anti-UFD1L	Proteintech	Cat# 10615-1-AP; RRID: AB_2213944
Rabbit polyclonal anti-53BP1	Novus Biologicals	Cat# NB100-304; RRID: AB_10003037
Rabbit monoclonal anti-Phospho-Histone H2A.X (Ser139) (20E3)	Cell Signalling	Cat##9718
Rabbit polyclonal anti-Lamin B1	Abcam	Cat# ab16048; RRID: AB_443298
Secondary donkey anti-rabbit Alexa Fluor 555	ThermoFisher Scientific	Cat# A-31572; RRID: AB_162543
Secondary goat anti-mouse (H+L DyLight™ 680 4X PEG Conjugate)	Cell Signaling Technology	Cat# 5470; RRID: AB_10696895
Secondary goat anti-mouse (H+L DyLight™ 800 4X PEG Conjugate)	Cell Signaling Technology	Cat# 5257; RRID: AB_10693543
Secondary goat anti-rabbit (H+L DyLight™ 800 4X PEG Conjugate)	Cell Signaling Technology	Cat# 5151; RRID: AB_10697505
Secondary goat anti-mouse (HRP-linked)	Cell Signaling Technology	Cat# 7076; RRID: AB_330924
Secondary goat anti-rabbit (HRP-linked)	Cell Signaling Technology	Cat# 7074; RRID: AB_2099233
**Bacterial and virus strains**		
BL21	New England Biolabs	C2530H
**Chemicals, peptides, and recombinant proteins**		
Polyethylenimine	Polysciences	Cat# 23966-1
Polybrene	Merck Millipore	Cat# TR-1003-G
Puromycin	Sigma-Aldrich	Cat# P8833
G418	Thermo Fisher Scientific	Cat# 10131035
Lipofectamine 3000	Thermo Fisher Scientific	Cat# L3000008
RNAiMAX	Thermo Fisher Scientific	Cat# 13778150
Dharmafect-1	Horizon Discovery	Cat# T-2001-02
Chlorophenol red-B-D-galactopyranoside (CPRG)	Sigma-Aldrich	Cat# 10884308001
Reversine	Sigma-Aldrich	Cat# R3904
SiR-actin	Spirochrome	Cat# CY-SC001
Hoechst 33258	Sigma-Aldrich	Cat# 861405
Isopropyl β-d-1-thiogalactopyranoside (IPTG)	Generon	Cat# IB0168-25G
Glutathione sepharose 4B beads	GE Healthcare Life Sciences	Cat# 17075601
cOmplete protease inhibitor cocktail	Sigma-Aldrich	Cat# 000000011697498001
GFP-Trap M	ChromoTek	Cat#GTM-20
Eeyarestatin I	Sigma-Aldrich	Cat# E1286
Kifunensine	Sigma-Aldrich	Cat# K1140
Epoxy resin	TAAB	Cat# T028
Uranyless stain	TAAB	Cat# S474
Reynolds lead citrate	TAAB	Cat# L037
Prolong Diamond Antifade Mountant	Thermo Fisher Scientific	Cat# P36965

(*Continued on next page*)

Continued		
REAGENT or RESOURCE	SOURCE	IDENTIFIER
(S)-3'-amino Blebbistatin	Cambridge Bioscience Ltd	Cat# CAY24170
Y-27632 dihydrochloride	Sigma-Aldrich	Cat# Y0503
Critical commercial assays		
ABI High-Capacity cDNA Reverse Transcription Kit	Thermo Fisher Scientific	Cat# 4368814
Experimental models: cell lines		
HT1080	ATCC	Cat# CRL-7951; RRID: CVCL_0317
HeLa	ATCC	Cat# CRM-CCL-2; RRID: CVCL_0030
HEK 293T	ATCC	Cat# CRL-3216; RRID: CVCL_0063
HT1080δLaminA/C	De Vos laboratory (Antwerp University)	N/A
Experimental models: organisms/strains		
Y190 Yeast Strain	Harper et al., 1993	N/A
Oligonucleotides		
siNT: ON-TARGETplus Non-targeting Control siRNA	Horizon Discovery (Dharmacon)	D-001810-01-50
siBROX: Hs_C1orf58_3 FlexiTube siRNA	Qiagen	SI04234300
siCHMP7: 5'-GGGAGAAG AUUGUGAAGUUdTdT-3'	Ventimiglia et al., 2018	Horizon Discovery (Dharmacon) Custom siRNA
siNesprin-2.1: 5'-GAGUGUCG GAGGGAACUAAUU-3'	Jayo et al., 2016	Horizon Discovery (Dharmacon) Custom siRNA
siNesprin-2.2: 5'-GAAGAAAAG GUGCAUGUUAUU-3'	Jayo et al., 2016	Horizon Discovery (Dharmacon) Custom siRNA
siUFD-1: 5'-GAGGCAGA UUCGUCGCUUUdTdT-3'	Olmos et al., 2015	Horizon Discovery (Dharmacon) Custom siRNA
Recombinant DNA		
pCMS28 mCherry-NLS	Ventimiglia et al, 2018	N/A
pCMS28 mCherry-BAF	This paper	N/A
pNG72 GFP-L-BROXr	This paper	N/A
pNG72 GFP-L-BROXr C408S	This paper	N/A
pCMS28 mCherry-Emerin	Ventimiglia et al, 2018	N/A
pNG72 CHMP4B-L-GFP	Ventimiglia et al, 2018	N/A
YFP-LAP2β	Carlton et al.,2012	N/A
pNG72 GFP-Emerin	This paper	N/A
pNG72 GFP-NLS	Ventimiglia et al, 2018	N/A
pGEX Empty	Agromayor et al. 2009	N/A
pGEX BROX WT	This paper	N/A
pGEX BROX L350A	This paper	N/A
pGEX BROX H204A	This paper	N/A
pCR3.1 GFP-CHMP4B	Ventimiglia et al, 2018	N/A
pCR3.1 GFP-empty	Ventimiglia et al, 2018	N/A
pCR3.1 hmN2G$^{SR29-33}$	This paper	N/A
pNG72 GFP-L-BROXr L350A	This paper	N/A
KT7 BROX WT	This paper	N/A
KT7 BROX E137A	This paper	N/A
KT7 BROX H144A	This paper	N/A
KT7 BROX R145A	This paper	N/A
KT7 BROX H204A	This paper	N/A
KT7 BROX L208A	This paper	N/A
KT7 BROX L208/212D	This paper	N/A

(*Continued on next page*)

Continued

REAGENT or RESOURCE	SOURCE	IDENTIFIER
KT7 BROX L212D	This paper	N/A
KT7 BROX K322A	This paper	N/A
KT7 BROX Y348A	This paper	N/A
KT7 BROX Y348A	This paper	N/A
KT7 BROX L350A	This paper	N/A
KT7 BROX I354A	This paper	N/A
HB18 CHMP4B	Martin-Serrano et al, 2003	N/A
HB18 CHMP5 C-terminal truncation	This paper	N/A
HB18 Nesprin-2	This paper	N/A
pNG72 GFP-SOUBA	This paper	N/A
HA-Ubiquitin	Agromayor and Martin-Serrano, 2006	N/A
pCR3.1 FLAG-BROX WT	This paper	N/A
pCR3.1 FLAG-BROX H204A	This paper	N/A
pCR3.1 GFP-hmN2G	This paper	N/A
pCR3.1 FLAG-BROX L350A	This paper	N/A
Software and algorithms		
FIJI	https://fiji.sc/	N/A
Prism 9	GraphPad	N/A
Imaris 9	Bitplane	N/A
AutoQuant X3	Media Cybernetics	N/A
UCSF Chimera	https://www.cgl.ucsf.edu/chimera/	N/A

RESOURCE AVAILABILITY

Lead contact

Further information and requests for resources and reagents should be directed to and will be fulfilled by the lead contact, Monica Agromayor (monica.agromayor@kcl.ac.uk)

Materials availability

Plasmids and cell lines generated in this study are available from the lead contact upon request.

Data and code availability

- All data reported in this paper will be shared by the lead contact upon request
- This paper does not report original code
- Any additional information required to reanalyse the data reported in this work paper is available from the lead contact upon request

EXPERIMENTAL MODEL AND SUBJECT DETAILS

Cell lines

Cells were cultured at 37°C and 5% humidified CO_2. All cells were grown in Dulbecco's Modified Eagle Medium (DMEM, Gibco, MA, USA) supplemented with 10% fetal bovine serum (FBS) (Sigma-Aldrich, MO, USA) and 20µg/mL gentamicin (Life Technologies MA, USA). Cells were regularly tested for the presence of mycoplasma. HT1080$^{\delta LMNA}$ cells were a kind gift from Professor Winnock De Vos (Antwerp University, Belgium)(Robijns et al., 2016).

METHOD DETAILS

Plasmids

Wild-type BROX was amplified by polymerase chain reaction (PCR) from cDNA (GenBank: NM_144695.3). BROX point mutations and siRNA resistant forms of BROX were generated using PCR site-directed mutagenesis. BROX constructs containing a 25 nm flexible linker (GGGGSx13) and human mini-Nesprin-2G (hmN2G) were created by gene synthesis (GeneWiz). hmN2G constructs were

designed to match previously described mini-Nesprin-2G constructs utilising the mouse nesprin-2 sequence(Luxton et al., 2010). hmN2G contains residues 3-482, two flexible linkers (GGGGSx2) flanking a NotI site followed by residues 6562-6907. hmN2G$^{SR29-33}$ adds the BROX binding region encompassing residues 1860-3790. The C-terminal fragment of human CHMP5 (residues 149–219) was amplified by PCR. Plasmids that express GAL4-BROX fusion proteins were derived by insertion of BROX encoding sequences into pGBKT7 (Clontech, Palo Alto, California). Plasmids that express CHMP4B, CHMP5149-219 and hmN2G$^{SR29-33}$ in yeast, fused to a VP16 activation domain and an HA tag, were derived by insertion of PCR products into pVP16/HA (Bogerd et al., 1993). The plasmid containing an HA-tagged form of ubiquitin has been described elsewhere (Treier et al., 1994). The list of plasmids used in this study is detailed in the key resources table.

Generation of stable cell lines

HT1080 and HeLa cells stably expressing fluorescently tagged fusion proteins were generated using MLV-based retroviruses as described previously(Ventimiglia et al., 2018). Briefly, 293T cells were transfected with 200 ng of pHIT-VSVG, 900 ng of MLV-GagPol, and 900 ng of the pCMS28 or pNG72 retroviral packaging vectors using polyethylenimine (PEI; Polysciences, Germany). Viral-containing supernatants were collected 48hr later, mixed with 8 μg/mL polybrene (Merck Millipore, Germany) before being and used to transduce target cells. Selection with puromycin (200 ng/ml, Sigma-Aldrich) or G418 (500 μg/ml, Invitrogen, ThermoFisher, MA, USA) was applied 48 hr later, and cells were passaged under continual selection. Cells stably expressing CHMP4B-L-GFP, mCherry-Emerin, mCherry-NLS, H2B-mCherry and YFP-Lap2β, have been described previously (Ventimiglia et al., 2018).

Generation of HT1080$^{\delta BROX}$ cell line

Guide RNAs targeting the sites located in the 5’and 3’ends of the BROX gene locus were designed using the Zhang Lab website (http://crispr.mit.edu) and cloned into px330-U6-Chimeric_BB-CBh-hSpCas9 plasmid (Addgene plasmid #42230). The sequences are as follows (5’F-CACCGTTTTGTCTATAGAAAACATC; 5’R- AAACGATGTTTTCTATAGACAAAAC; 3’F-CACCGTCAAACCTCAA AAGGACACT; 3’R-AAACAGTGTCCTTTTGAGGTTTGAC). HT1080 cells were co-transfected with both guide RNA containing plasmids and a pCR3.1-GFP plasmid (2.5μg of each guide RNA plasmid and 25ng of pCR3.1-GFP) using Lipofectamine 3000 (Invitrogen). After 48 hours, single cells expressing GFP were sorted using FACS (BD FACS Aria III, BD, NJ, USA) and recovered in DMEM supplemented with 20% FCS to facilitate cell growth. Successful clones were then analysed for BROX knockout by western blotting to confirm the loss of the endogenous protein.

Transfections

For transient expression of GFP-, HA- or FLAG-tagged proteins in 293T or HT1080 cells, transfections were performed using PEI. Cells were grown on 6-well plates for 24 hours to reach 70% confluency before transfection with the appropriate plasmids (to a maximum of 2 μg DNA) using 16 μL PEI. Cells were washed after 16 hours and harvested 36 hours post transfection.

For siRNA transfections, HT1080 were plated and transfected concurrently with siRNA to a final concentration of 10 nM using RNAiMAX (Invitrogen) according to manufacturer’s instructions. 48 hours post transfection the cells received a second round of siRNA at the same concentration. Cells were either imaged or fixed 16h post transfection. For partial depletion of Nesprin-2, HT1080 cells were transfected once with 5 nM each of custom Nesprin-2 specific oligos Nesprin-2.1 and Nesprin-2.2 for final concentration of 10 nM. HeLa cells were transfected with 100 nM of the corresponding siRNA using Dharmafect-1 (Dharmacon RNA technologies, CO, USA) according to manufacturer’s instructions. A list of siRNAs used is provided in the key resources table. Protein depletion was confirmed by western blotting or qPCR as described below.

Western blotting

Samples were denatured in Laemmli buffer before resolving via SDS-PAGE. Proteins were then transferred onto 0.2 μM nitrocellulose membranes (Protran, GE Healthcare) probed with the indicated primary and secondary antibodies diluted in 1% milk. A list of antibodies used is provided in the key resources table. For HRP-conjugated secondary antibodies, membranes were further treated with Amersham ECL Prime western blotting detection reagent (GE Healthcare). Membranes were visualised using Li-Cor Odyssey Infrared scanner and software.

qPCR

HT1080 cells were plated onto 24-well plates at a density of $3x10^4$ and transfected with siRNA as described above. 24 hours post transfection, cells were harvested for RNA extraction using the RNeasy Mini Kit (Qiagen) following manufacturer’s instructions. In brief, cells were directly lysed in the plate by addition of the lysis buffer and homogenized by syringing. RNA was then purified by passing through spin columns and washing. Purified RNA then underwent reverse transcription PCR using the ABI High Capacity cDNA Reverse Transcription Kit (ThermoFisher) following manufacturer’s instructions and using the following thermocycler settings: 25 °C 10 min; 37 °C 120 min; 85 °C 5 min; 4 °C hold. qPCR was performed using the TaqMan Fast Advanced Master Mix kit according to manufacturer’s instructions with the following probes; nesprin-2 (Hs00396027_m1) (TaqMan Gene EX Assays, ThermoFisher), GAPDH (Hs99999905_m1) (TaqMan Gene EX Assays, ThermoFisher).

Yeast-two hybrid assays

Genome-wide screen by Hybrigenics

Full-length BROX cloned in pB27 (N-LexA-bait-C fusion) was used in an ULTImate Y2H screen against the Human Placenta_RP5 complementary DNA Gal4-activating domain-fusion library (Hybrigenics, Paris, France). Prey fragments of positive clones were amplified by PCR and sequenced at their 5′ and 3′ junctions and the resulting sequences were used to identify corresponding interacting proteins in the GenBank database via a fully automated procedure. Hybrigenics automatically computed through their algorithms a predicted biological score (PBS) for each interaction to assess the reliability of the interaction and ranked the results, depending on the confidence of binding in four categories A to D (A having the highest confidence of binding).

Yeast two-hybrid alanine scanning

Yeast cells (Y190)(Harper et al., 1993) were transformed with the pGBKT7 and pVP16/HA-derived plasmids described above and grown for 3 days at 30C on Sabourard agar (SD, Sigma-Aldrich) supplemented with Leucine-Tryptophan drop-out medium (Sigma-Aldrich) to select for positive co-transformants. Protein-protein interaction was screened by β-galactosidase activity using chlorophenol red-β-D-galactopyranoside (CPRG, Roche, Switzerland). Data is represented as the mean ± SEM of three technical repeats from one representative experiment out of three independent assays and β-gal activities are expressed as absorbance units (A540 nm); the background activity in these assays (using pGBKT7 and pVP16/HA transformants) is approximately 0.01 absorbance units.

Analysis of BROX conservation and 3D modelling

Electrostatic surface representations of BROX Bro1 domain are based on the previously described structure (PDB: 3ULY)(Mu et al., 2012) The analysis of BROX conservation across multiple species was performed using the ConSurf server(Ashkenazy et al., 2016). 3D surface modelling of BROX was achieved with UCSFChimerau(Pettersen et al., 2004; Sanner et al., 1996) sing the previously described structure.

Mechanical perturbation of cells using an AFM

HT1080 wild-type or HT1080$^{\delta BROX}$ cells stably expressing GFP-NLS and H2B-mCherry were seeded on glass-bottom dishes (WillCo Wells) and starved for 24 hours. Experiments were performed using a Bruker BioScope Resolve AFM. Silicon nitride cantilevers, with a nominal spring constant of 0.6 N/m, with four-sided pyramidal tips, with semi-included front, back, and side angles of 35°, nominal tip radius of 20 nm, and minimal tip height of 3.2 μm, were used (MLCT-Bio-DC Tip F, Bruker). These probes are specifically designed to have a low thermal sensitivity to minimize the thermal influence of fluorescent excitation. The spring constant was calibrated by thermal tuning in MilliQ water with the simple harmonic oscillator model fit in the NanoScope. During acquisition, cells were maintained at 37 °C with a combination of stage and objective heaters, with the medium buffered with 50 mM HEPES. The Resolve AFM is incorporated with a Nikon Eclipse Ti inverted epifluorescence microscope with a 60x NA 1.40 oil immersion objective and a CoolLED illumination source. Cell nuclei were identified, and a constant force (100 nN, using z-feedback control) was applied perpendicular to the cell nucleus for 5 minutes. GFP-NLS expression was used to monitor nuclear envelope integrity, which was confirmed by nuclear localization of this fluorescent signal. Images were acquired every 10 seconds for 10 minutes using an Andor iXon Ultra 888. To avoid mechanical noise of filter cube turret rotation, an OptoSplit II Bypass Image Splitter (Cairn Research), was implemented, allowing the simultaneous acquisition of two different optical wavelengths images (GFP-NLS and H2B- mCherry) on either side of the camera sensor.

Breakthrough forces experiments

Force spectroscopy measurements on cells using high-indenting forces were previously described (Atilla-Gokcumen et al., 2014). Briefly, cells were plated on cell culture glass-bottom dishes (WillCo Wells) 24h prior the AFM experiments. Experiments were performed using a commercial Bruker BioScope Resolve AFM coupled with a Nikon Eclipse Ti inverted epifluorescence microscope. Cells were maintained at 37 °C with a combination of stage and objective heaters and the medium was buffered with 50 mM HEPES.

MLCT tip F, k = 0.6 N/m, Bruker AXS, Karlsruhe, Germany, were calibrated as described before, by thermal tuning in MilliQ water with the simple harmonic oscillator model fit in the NanoScope. Optical microscopy was used to localise the AFM tip on the top of the cell cytoplasm or nucleus. To avoid overlapping regions from neighbouring cells, only isolated cells were indented. Cells were indented at a constant velocity of 1μm sec-1 until the culture dish substrate was encountered (applying forces up to 120 nN).

The penetration and rupture of individual membranes within the cell by the AFM probe were observed in the discontinuity of the force versus extension trace and fingerprinted by a series of breakthrough jumps (Yokokawa et al., 2008). Traces showing two breakthrough events indicate the probe has indented the plasma membrane, whereas traces showing more than four breakthrough jumps indicate that the probe has punctured both the plasma membrane and the NE.

Coprecipitation assays

Recombinant protein pulldowns

Competent BL21.DE3 E. coli bacteria were transformed with pGEX vectors expressing WT BROX or BROX mutants and grown in 250 mL lysogeny broth (LB) media (Sigma-Aldrich). Isopropyl β-d-1-thiogalactopyranoside (IPTG, Generon, UK) was added after reaching an OD600 of 0.8 to a final concentration of 0.5 mM and left overnight at 16°C to induce protein expression. Purification of recombinant proteins was performed at 4°C. Bacteria was then collected and resuspended in GEX-lysis buffer (50 mM Tris,

pH 7.4, 100 mM NaCl, 1 mM EDTA, lysozyme 1 mg/ml and a protease inhibitor cocktail (complete mini-EDTA free, Sigma-Aldrich) before sonication and clarified protein was collected by centrifugation at 15,000g for 90 minutes. Clarified proteins were purified using glutathione sepharose 4B beads (GE Healthcare, IL, USA) pre-equilibrated in lysis buffer for 3 hours before washing. 293Ts cells were simultaneously transfected with the indicated pCR3.1 GFP tagged fusion proteins (500 ng GFP-empty, 1000 ng GFP-CHMP4B, 1300 ng GFP-hmN2G$^{SR29-33}$). Cells were harvested 36 hours post transfection and lysed in GST-lysis buffer (50 mM Tris, pH 7.4, 150 mM NaCl, 5 mM EDTA, 5% glycerol, 1% Triton X-100 and a protease inhibitor cocktail (complete mini-EDTA free, Sigma). Clarified lysates were incubated with recombinant BROX proteins bound to glutathione sepharose 4B beads for 3h at 4°C before washing (50 mM Tris, pH 7.4, 100 mM NaCl, 1 mM EDTA). Bound proteins were eluted by boiling samples in 100 uL of Laemmli buffer.

GFP immunoprecipitations

293T cells transiently co-expressing GFP-hmN2G or GFP-hmN2G$^{SR29-33}$, and HA-ubiquitin were lysed at 4°C in 1 ml of lysis buffer (50 mM Tris, pH 7.4, 150 mM NaCl, 5 mM EDTA, 5% glycerol, 1% Triton X-100 and a protease inhibitor cocktail (complete mini-EDTA free). The lysates were sonicated and centrifugated at 15,000 rpm for 10 minutes at 4°C and the clarified supernatant were incubated with anti-GFP coupled magnetic microparticles (GFP-Trap, ChromoTek, Germany) for 2 hours. The microparticles were washed four times with wash buffer (50 mM Tris, pH 7.4, 150 mM NaCl, 5 mM EDTA, 5% glycerol, and 0.1% Triton X-100). Bound proteins were eluted in 60 μl of Laemmli buffer, boiled and analyzed by western blot. Eeyarestatin I (2 μg/ml, Sigma-Aldrich) or Kifunensine (10 μM, Sigma-Aldrich) were added 16 hours before lysis when required.

Flow cytometry

HT1080 cells stably expressing GFP-hmN2G$^{SR29-33}$ were transfected with the corresponding siRNA as indicated above. 24 hours after the second round of siRNA transfection, cells were resuspended in FACs buffer (1% FCS in PBS) and analysed for GFP expression using a BD FACSCanto II flow cytometer (BD). Flow-cytometry data was analysed with FlowJo software (TreeStar).

Transmission Electron Microscopy (TEM)

For transmission electron microscopy (TEM) analysis, cells were growth on glass coverslips at a confluency of 10x104 cells and fixed overnight at 4 °C in 2.5% Glutaraldehyde/2% Paraformaldehyde in 0.1M Cacodylate buffer (pH 7.4). After fixation, cells were washed briefly with 0.1M cacodylate buffer (pH 7.4) and post-fixed in 1% (v/v) osmium tetroxide in cacodylate buffer. Then, cells were en-bloc stained with 1% (w/v) aqueous uranyl acetate, thoroughly washed and dehydrated through a graded ethanol series before infiltration with epoxy resin (T028, TAAB, UK). Finally, cells were flat embedded and polymerise at 60 °C for 24h. Ultrathin sections (70-80nm) were cut using an ultramicrotome (UC 7, Leica microsystems, Germany), mounted on grids and post-stained with Uranyless stain and Reynolds lead citrate (TAAB).

Samples were examined using a TEM operated at 120Kv (JEOL JEM 1400Plus, JEOL, Japan). Images were acquired with a 2k by 2k format CCD camera (JEOL Ruby CCD Camera, JEOL, Japan).

Immunofluorescence

HT1080 cells were grown on coverslips and fixed for 20 minutes with methanol at -20 °C or 4%PFA at room temperature, depending on primary antibody used. Cells were blocked in PBS containing 1% FBS for 1 hour followed by incubation with primary antibodies for 3 hours. After washing, cells were then stained with Alexa Fluor-conjugated secondary antibodies (Thermo Fisher Scientific). In conjunction, DNA was stained with Hoechst 33258 (Sigma-Aldrich). Coverslips were finally mounted onto slides using ProLong Diamond Antifade Mountant (Thermo Fisher Scientific).

Fixed cells were imaged using either a Nikon Eclipse Ti wide-field inverted microscope equipped with a CoolSnap HQ2 CCD camera (Photometrics, AZ, USA) or a Nikon Eclipse Ti inverted CSU-X1 spinning disk confocal microscope (Nikon, Japan) equipped with an Andor Zyla VSC-10899 (Andor, UK).

QUANTIFICATION AND STATISTICAL ANALYSIS

Image analysis

All microscopy analysis was done blind.

NERDI analysis in unconstrained migration

HT1080 cells stably co-expressing mCherry-NLS and the corresponding GFP-fused BROX proteins were grown on glass bottomed 24-well plates (Eppendorf, Germany) and transfected with the appropriate siRNA. An hour before imaging, cells were treated with Hoechst 33258 (1:1000) to stain DNA and consistently track nuclei in successive time frames. Cells were imaged lived for 16 hours using a 60x oil-immersion objective (NA 1.4) equipped Nikon Eclipse Ti inverted CSU-X1 spinning disk confocal microscope attached to an environmental chamber. Images were acquired every 5 minutes using an Andor Zyla VSC-10899 camera with 2x2 binning.

NERDI events were identified as abrupt drops in mCherry-NLS nuclear fluorescence intensity. Because cytoplasmic accumulation of mCherry-NLS indicates NE rupture, and repair is indicated by its re-accumulation in the nucleus, mCherry signal was quantified over time by drawing a small region of interest (ROI) both in the nucleus and cytoplasm and intensities were extracted using FIJI (Schindelin et al., 2012). The ratio of both signal intensities, expressed as arbitrary units (a.u.), was used to evaluate the rupture repair times. This considers differences in expression levels of mCherry-NLS for each cell. These values were then normalized to the

mCherry-NLS intensity of the frame prior to nucleus rupture and the first drop in mCherry-NLS intensity (rupture start) set to T=0. Recovery time was calculated by counting the number of frames it takes for the nucleo/cytoplasmic ratio to reach at least 0.9 a.u. and multiplying by the image acquisition frequency. To increase the frequency of NERDI events in this setting, cells were treated 16 hours before imaging with 0.1 μM of the MPS1 kinase inhibitor reversine (Sigma-Aldrich), which causes abnormalities in chromosome segregation during mitosis that produce chromosome bridges and predisposes the NE to rupture (Maciejowski et al., 2015).

NERDI analysis using confinement microchannels

HT1080 or HT1080$^{\delta BROX}$ cells stably co-expressing mCherry-NLS and the corresponding GFP-tagged proteins were seeded into 6-well plates and transfected with the appropriated siRNA. After 48 hours, cells were resuspended in 60 μL DMEM and seeded in RTV 615 clear silicone (Momentive Performance, NY, USA) microchannels bearing 4μm constrictions previously mounted on 35mm glass-bottom plates (Ibidi, Germany). Fresh media was added to just cover the bottom of the channels. 6 hours later, fresh media supplemented with Hoechst 33258 (1:1000) to stain DNA was added to fully submerge the channels for 1 hour before imaging live for 16 hours using a 60x oil-immersion objective (NA 1.4) equipped Nikon Eclipse Ti inverted CSU-X1 spinning disk confocal microscope with attached environmental chamber. Z-series (1.5 μm step size) images were acquired every 5 minutes using an Andor Zyla VSC-10899 camera. For NERDI analysis, rupture events were identified as abrupt drops in mCherry-NLS nuclear fluorescence intensity in cells migrating through constrictions and were analysed as described above.

Quantification of 53BP1 and γH2AX foci

HT1080 WT, δBROX or δBROX GFP-L-BROX^r cells were grown on coverslips, fixed and stained with with either an α-53BP1 or an α-γH2AX antibody and Hoescht as described above. Maximum intensity projects of whole cell Z-stack images were used to identify nuclei by automated thresholding of the Hoescht signal before establishing a threshold for foci using the parental HT1080 cells. The number of foci per nucleus were automatically counted using the 'Analyse Particles' tool of FIJI (Schindelin et al., 2012) with a minimum particle size of 0.1.

Quantification of actin cables

HeLa, HT1080 and HT1080$^{\delta BROX}$ cells stably expressing YFP-Lap2β were plated onto glass-bottomed 24-well plates at a density of 2.5x10^4 cells per well. Cells were transfected with the appropriate siRNA as described above. Before imaging, cells were incubated with silicon-rhodamine actin (SiR-actin, Spirochrome, CO, USA) at a final concentration of 100 nM for 60 minutes. For myosin-II inhibition experiments, cells were treated with 50 μM (S)-3'-amino Blebbistatin (Cambridge Bioscience Ltd) or 50 μM Y27632 (Sigma-Aldrich) for 1 hour before addition of Sir-actin. Z-series (0.3 μm step size) were captured live using either a Nikon Eclipse Ti wide-field inverted microscope equipped with a CoolSnap HQ2 CCD camera (for HeLa cells); or a Nikon Ti-eclipse inverted CSU-X1 spinning disk confocal microscope using an Andor Zyla VSC-10899. Images were blinded before analysis and fields with sparse or isolated cells were chosen to aid in identifying individual cells. Actin cables were defined as linear arrays of actin crossing the dorsal surface of the nucleus and coinciding with a Lap2β positive groove.

Quantification of NE invaginations and intranuclear tubules (INTs)

For analysis of NE morphology, cells were plated on coverslips, fixed, and stained with an α-Lamin B1 antibody as described above. Coverslips were imaged blind and presence of NE invaginations was quantified by eye based on the presence of lamina folds at the NE periphery that were present in multiple z-stacks.

For quantification of INTs, HT1080 and HT1080$^{\delta BROX}$ cells stably expressing either GFP-Emerin or mCherry-Emerin plus GFP-L-BROX^r constructs were plated onto 24-well glass bottomed plates at a density of 2.5x10^4, treated with the appropriate siRNA as described above, and imaged live. The entire volume of the cells was captured as a Z-series with 0.1 μm step size using a 100x oil-immersion objective (NA 1.45) equipped Nikon Eclipse Ti inverted CSU-X1 spinning disk confocal microscope with attached environmental chamber and an Andor Zyla VSC-10899 camera. INTs were quantified using the volumetric view in the Nikon Advanced Research Program (4.1) (Nikon). 3D reconstructions were performed in Imaris (Bitplane, UK) using the surfaces function.

Quantification of fluorescence intensity profiles

HT1080 cells stably co-expressing mCherry-Emerin along with GFP-L-BROXr WT or C408S were seeded on glass bottomed 24-well plates and transfected with BROX-specific siRNA oligos. Live cells were imaged with a 100x oil-immersion objective equipped (NA 1.45) Nikon Eclipse Ti CSU-X1 inverted spinning disk confocal microscope using an Andor Zyla VSC-10899 camera. The entire volume of the cells was captured as a Z-series with 0.1 μm step size before deconvolution using AutoQuant X3 Deconvolution Software (Media Cybernetics, MD, USA). mCherry-Emerin and GFP-L-BROXr fluorescence was quantified using Nikon Advanced Research Program (4.1) by drawing a line through the nuclear envelope and plotting the data in Microsoft Office Excel (Microsoft, WA, USA)

Analysis of perinuclear GFP-SOUBA accumulation and nuclear rounding quantification

HT1080 cells stably co-expressing mCherry-NLS and GFP-SOUBA were grown, transfected and imaged as described above. Changes in nuclear roundness over time were quantified using FIJI (Schindelin et al., 2012) In brief, Hoechst 33258 positive nuclei were used to generate a nucleus specific region of interest (ROI) which could then be measured for roundness. This step could then be repeated for each timeframe for the duration of the movie.

Quantification of hmN2G$^{SR29-33}$ cables and cell death

HT1080 and HT1080$^{\delta BROX}$ cells stably expressing GFP-hmN2G$^{SR29-33}$ and mCh-NLS were seeded in RTV 615 clear silicone (Momentive Performance, NY, USA) microchannels bearing 4μm constrictions and imaged lived as described above. For the quantification of hmN2G$^{SR29-33}$ cables, accumulation of GFP signal was identified in all z-stacks as cells migrated through the constriction and NE rupture was determined based on nuclear mCherry-NLS signal loss. Cell death was determined visually by morphological changes such as appearance of nuclear fragments, loss of nuclear compartmentalisation and cytoplasm shrinking. Dead cells were counted if they had migrated through the channel and died within 1 hour of exiting the constriction. Panel 4I includes cells that die in panel 4J.

Statistical analysis

Graphs and statistical tests were made using GraphPad Prism 9 (GraphPad, CA, USA). In all cases, error bars are mean ±SEM. Statistical tests used were two-tailed unpaired t-tests, Mann-Whitney test or one-way ANOVA with Tukey post-hoc multiple comparisons test where appropriate. All experiments are representative of at least 3 biological replicates. Details of statistical significance and information about sample size are included in their respective figure legends. $^{N.S}p>0.05$ represents no significant difference. Statistical difference is shown as *$p<0.05$, **$p<0.01$, ***$p<0.001$ and ****$p<0.0001$. For curves shown in Figures 1A, 1G, 3F, 3K, and 4B, T=0 marks the rupture start and the significance compared to the control was calculated at 35 min using a two-tailed unpaired t test.

Developmental Cell, Volume *56*

Supplemental information

The ESCRT machinery counteracts Nesprin-2G-mediated mechanical forces during nuclear envelope repair

Samuel S. Wallis, Leandro N. Ventimiglia, Evita Otigbah, Elvira Infante, Miguel Angel Cuesta-Geijo, Gururaj Rao Kidiyoor, M. Alejandra Carbajal, Roland A. Fleck, Marco Foiani, Sergi Garcia-Manyes, Juan Martin-Serrano, and Monica Agromayor

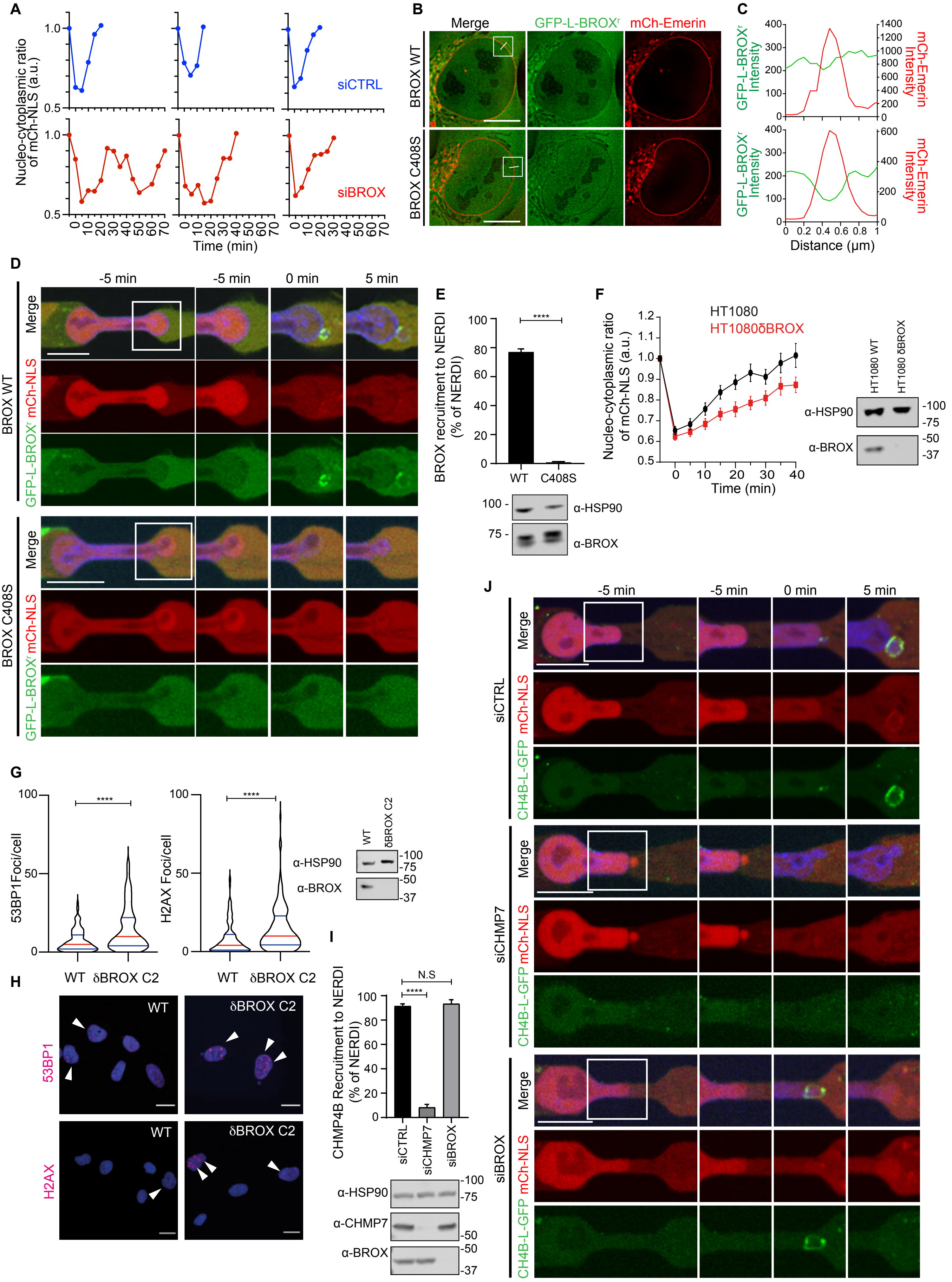

A
Nucleo-cytoplasmic ratio of mCh-NLS (a.u.)
siCTRL
siBROX
Time (min)
B
Merge
GFP-L-BROXʳ
mCh-Emerin
BROX WT
BROX C408S
C
GFP-L-BROXʳ Intensity
mCh-Emerin Intensity
Distance (µm)
D
-5 min
0 min
5 min
Merge
mCh-NLS
GFP-L-BROXʳ
BROX WT
BROX C408S
E
BROX recruitment to NERDI (% of NERDI)
WT
C408S
α-HSP90
α-BROX
F
HT1080
HT1080δBROX
Nucleo-cytoplasmic ratio of mCh-NLS (a.u.)
Time (min)
HT1080 WT
HT1080 δBROX
α-HSP90
α-BROX
G
53BP1Foci/cell
H2AX Foci/cell
WT
δBROX C2
α-HSP90
α-BROX
H
53BP1
H2AX
WT
δBROX C2
I
CHMP4B Recruitment to NERDI (% of NERDI)
N.S
siCTRL
siCHMP7
siBROX
α-HSP90
α-CHMP7
α-BROX
J
-5 min
0 min
5 min
Merge
mCh-NLS
CH4B-L-GFP
siCTRL
siCHMP7
siBROX

Figure S1: BROX requires farnesylation for NE localisation and does not recruit CHMP4B to NE rupture sites. Related to Figure 1.

(A) Trace analysis of representative individual ruptures of similar size from siCTRL and siBROX treated cells and included in Figure1A.

(**B-C**) Analysis of BROX localisation in live HT1080 cells expressing mCherry-Emerin and GFP-L-BROX^r wild-type (WT) or non-farnesylated mutant (C408S) (B) Deconvolved images corresponding to the quantifications shown in (C). White lines mark the region of the NE where the fluorescence intensity profile was quantified. Scale bar, 10µm. (C) Fluorescence intensity profiles of HT1080 GFP-L-BROX^r WT (top) or C408S (bottom) cells.

(D-E) Time-lapse analysis of HT1080 cells expressing mCherry-NLS and GFP-L-BROX^r WT or C408S migrating through 4µm constrictions after treatment with siBROX. Hoechst was added to stain DNA. (D) Representative images of the quantifications shown in (E). Scale bar, 10µm. (E) Percentage of BROX-decorated NERDIs. GFP-L-BROX^r WT n=80; GFP-L-BROX^r C408S n=81; $p<0.0001$. Cell lysates were examined by blotting with the indicated antibodies.

(F) Recovery kinetics of NE integrity after NERDI in HT1080 wild-type (WT; n=29) or BROX-depleted (δBROX; n=46) cells stably expressing mCherry-NLS. Cell lysates were examined by blotting with the indicated antibodies.

(G-H) Analysis of DNA damage in HT1080 wild-type (WT) and BROX-depleted clone2 (δBROX C2) cells stained with α-53BP1 or α–γH2AX antibodies and Hoechst. (G) Quantification of 53BP1 and γH2AX -positive foci. The samples used for HT1080 WT were the same as in Figure 1I. WT n=147; HT1080$^{\delta BROX}$C2 n=147; $p<0.0001$. Median and quartiles are shown in red and blue respectively and cell lysates were examined by blotting with the indicated antibodies. (H) Representative images of the quantifications shown in (G). Arrowheads show DNA damage foci. Scale bar, 15µm.

(I-J) Time-lapse analysis of HT1080 cells expressing mCherry-NLS and CHMP4B-L-GFP. Cells were transfected with indicated siRNAs and imaged while migrating through 4µm constrictions. Hoechst was added to stain DNA. (I) Percentage of CHMP4B-decorated NERDI. siCTRL n=238; siCHMP7 n=81; siBROX n=137; ****$p<0.0001$, $^{N.S}p>0.05$. Cell lysates were analysed by blotting with the indicated antibodies. (J) Representative images of the quantification shown in (I). Scale bar, 10µm.

Figure S2

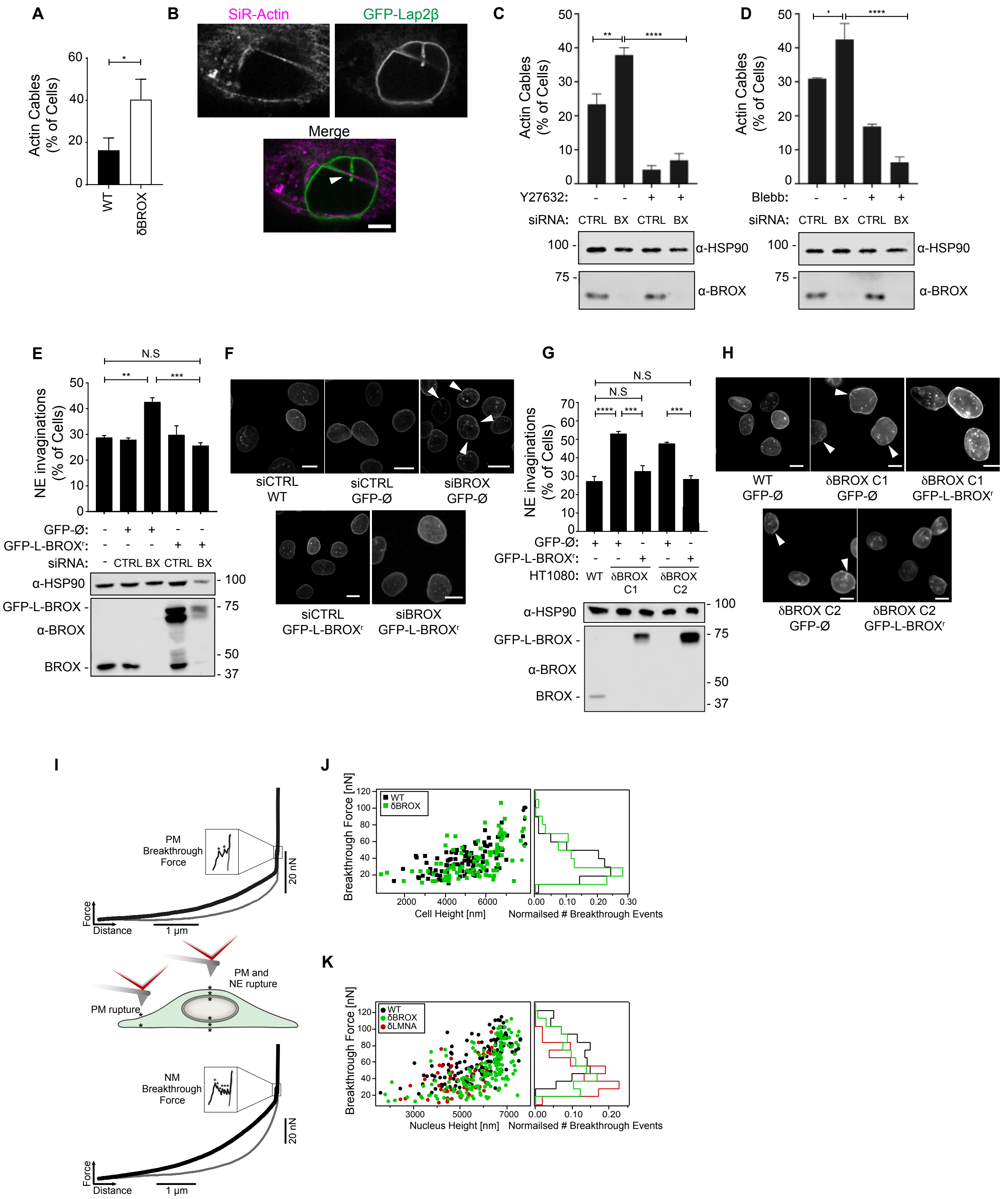

A
B
C
D
Actin Cables (% of Cells)
WT
δBROX
SiR-Actin
GFP-Lap2β
Merge
Y27632:
Blebb:
siRNA:
CTRL
BX
α-HSP90
α-BROX
E
F
G
H
NE invaginations (% of Cells)
N.S
GFP-Ø:
GFP-L-BROXʳ:
BROX
GFP-L-BROX
siCTRL WT
siCTRL GFP-Ø
siBROX GFP-Ø
siCTRL GFP-L-BROXʳ
siBROX GFP-L-BROXʳ
HT1080:
δBROX C1
δBROX C2
WT GFP-Ø
δBROX C1 GFP-Ø
δBROX C1 GFP-L-BROXʳ
δBROX C2 GFP-Ø
δBROX C2 GFP-L-BROXʳ
I
J
K
PM Breakthrough Force
20 nN
Force
Distance
1 μm
PM and NE rupture
PM rupture
NM Breakthrough Force
Breakthrough Force [nN]
Cell Height [nm]
Normalised # Breakthrough Events
Nucleus Height [nm]
δLMNA

Figure S2: NE phenotypes resulting from BROX depletion can be rescued by re-expression of GFP-L-BROX^r. Related to Figure 2.

(**A-D**) Analysis of actin cables in HT1080 wild-type (WT) or BROX-depleted (δBROX) stably expressing GFP-Lap2β and stained with SiR-actin. (A) Percentage of cells with nuclear actin cables. WT: n=160; δBROX n=116; p=0.0186. (B) Representative images of the quantifications shown in (A). Arrowhead denotes an actin cable. Scale bar, 5μm. (C-D) Percentage of cells with nuclear actin cables in cells treated with Y27632 or blebbistatin. siCTRL+H_2O: n=675; siBROX+H_2O n=611; siCTRL+Y27632: n=577; siBROX+Y27632 n=576; siCTRL+DMSO: n=680; siBROX+DMSO n=670; siCTRL+Blebb: n=570; siBROX+Blebb n=656; *p=0.0464, **p= 0.0068, ****p<0.0001

(**E-F**) Analysis of nuclear morphology in HT1080 cells stably expressing either GFP (GFP-ø) or GFP-L-BROX^r after treatment with the indicated siRNAs and staining with α-Lamin B1 antibody. (E) Quantification of percentage of cells with NE invaginations. Non-transfected n=559; GFP-ø+siCTRL n=487; GFP-ø+siBROX n=523; GFP-L-BROX^r+siCTRL n=667; GFP-L-BROX^r+siBROX n=721; **p=0.0031, ***p=0.0007, $^{N.S}$p>0.05. Cell lysates were analysed by blotting with the indicated antibodies. (F) Representative images of quantifications shown in (E). Arrowheads indicate NE invaginations. Scale bars, 10μm.

(**G-H**) Analysis of nuclear morphology in HT1080 WT and BROX-depleted cells (δBROX C1 and δBROX C2) stably expressing either GFP-ø or GFP-L-BROX^r after staining with α-Lamin B1 antibody. (G) Quantification of percentage of cells with NE invaginations. WT+GFP-ø n=246; δBROX C1+GFP-ø n=283; δBROX C1+GFP-L-BROX^r n=199, δBROX C2+GFP-ø n=291; δBROX C2+GFP-L-BROX^r n=270; ****p<0.0001, ***p<0.001, $^{N.S}$p>0.05. Cell lysates were analysed by blotting with the indicated antibodies. (H) Representative images of quantifications shown in (G). Arrowheads indicate nuclear NE invaginations. Scale bar, 10μm.

(I) Typical force-extension curves conducted on a live cell when only the plasma membrane (PM; top) or also the nuclear membrane (NM; bottom) are indented. Rupture of the plasma membrane (top inset) is hallmarked by two consecutive discontinuities in the contact region of the force-extension trace, while NE rupture can be fingerprinted by the presence of up to six discontinuities, corresponding to the rupture of the plasma membrane (two) in addition to the four nuclear membranes (bottom inset).

(J-K) Scatterplot of the breakthrough force versus the cell height in the PM or the NM. Histograms display the distribution of breakthrough forces corresponding to the rupture of the PM (J) or the NM (K). The colour-code corresponds to the type of cell analysed: WT (black), δBROX (green) and δLMNA (red). Number of traces are the same as in Figure 2I.

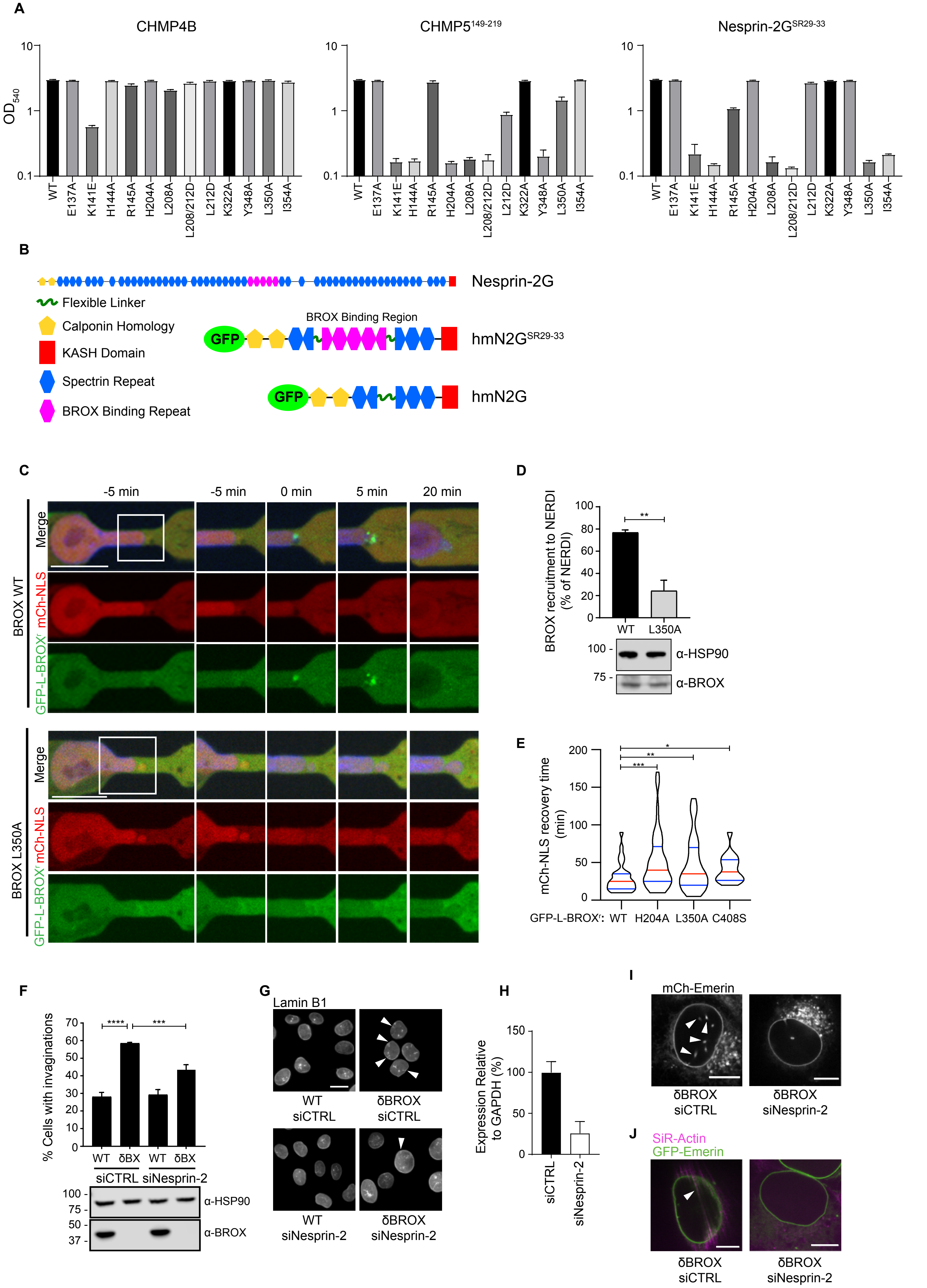
A
CHMP4B
CHMP5149-219
Nesprin-2GSR29-33
OD540
WT E137A K141E H144A R145A H204A L208A L208/212D L212D K322A Y348A L350A I354A
B
Nesprin-2G
Flexible Linker
Calponin Homology
KASH Domain
Spectrin Repeat
BROX Binding Repeat
BROX Binding Region
GFP
hmN2GSR29-33
hmN2G
C
-5 min
0 min
5 min
20 min
Merge
mCh-NLS
GFP-L-BROXr
BROX WT
BROX L350A
D
BROX recruitment to NERDI (% of NERDI)
WT
L350A
α-HSP90
α-BROX
E
mCh-NLS recovery time (min)
GFP-L-BROXr: WT H204A L350A C408S
F
% Cells with invaginations
WT δBX WT δBX
siCTRL siNesprin-2
α-HSP90
α-BROX
G
Lamin B1
WT siCTRL
δBROX siCTRL
WT siNesprin-2
δBROX siNesprin-2
H
Expression Relative to GAPDH (%)
siCTRL
siNesprin-2
I
mCh-Emerin
δBROX siCTRL
δBROX siNesprin-2
J
SiR-Actin
GFP-Emerin
δBROX siCTRL
δBROX siNesprin-2

Figure S3: Characterisation of BROX interactions and dependence on Nesprin-2G interaction for localisation during NERDI. Related to Figure 3.

(A) BROX was tested for interactions with CHMP4B (left graph), $CHMP5^{149\text{-}219}$ (middle graph) or Nesprin-$2G^{SR29\text{-}33}$ (right graph) by yeast two-hybrid assays.

(B) Diagram schematic of human mini-Nesprin-2G (hmN2G) constructs used. hmN2G is based on the previously published mN2G but uses the human sequence of Nesprin-2G. $hmN2GSR^{29\text{-}33}$ contains the BROX interacting region identified in the yeast-two hybrid screen flanked by flexible linkers.

(C-D) Time-lapse analysis of HT1080 cells expressing mCherry-NLS and GFP-L-$BROX^{r}$ wild-type (WT) or L350A mutant cells migrating through 4µm constrictions. Hoechst was added to stain DNA. (C) Representative images of quantifications shown in (D). Scale bar, 10µm. (D) Percentage of BROX-decorated NERDI. GFP-L-$BROX^{r}$ WT n=80; GFP-L-$BROX^{r}$ L350A n=113; *p=0.0058. The dataset corresponding to GFP-L-$BROX^{r}$ WT is the same used in the quantification shown in Figure S1E. Cell lysates were examined by blotting with the indicated antibodies.

(**E**) Quantification of the rupture recovery times in HT1080 cells expressing mCherry-NLS and GFP-L-$BROX^{r}$ wild-type (WT) or mutant forms (H204A, L350A, C408S); ***P=0.0007, **P=0.0067, *P=0.0391.

(F-G) Analysis of nuclear morphology in HT1080 wild-type (WT) or BROX-depleted (δBROX) cells transfected with indicated siRNAs and stained with α-Lamin B1 antibody. (F) Percentage of cells with NE invaginations. WT+siCTRL n=245; WT+siNesprin-2 n=168; δBROX+siCTRL n=176; δBROX+siNesprin-2 n=193; ****P<0.0001, ***P<0.001. Cell lysates were analysed by blotting with the indicated antibodies. (G) Representative images of quantifications shown in (F). Arrowheads indicate NE invaginations. Scale bar, 20µm.

(H) HT1080 cells transfected with either control or Nesprin-2-specific siRNA were analysed for Nesprin-2 mRNA levels by qPCR. Results were normalised to GAPDH mRNA levels.

(I) Representative images of quantifications in Figure 3H. Arrowheads indicate INTs. Scale bar, 10µm.

(J) Representative images of quantifications in Figure 3I. Arrowhead indicates an actin cable. Scale bar, 10µm.

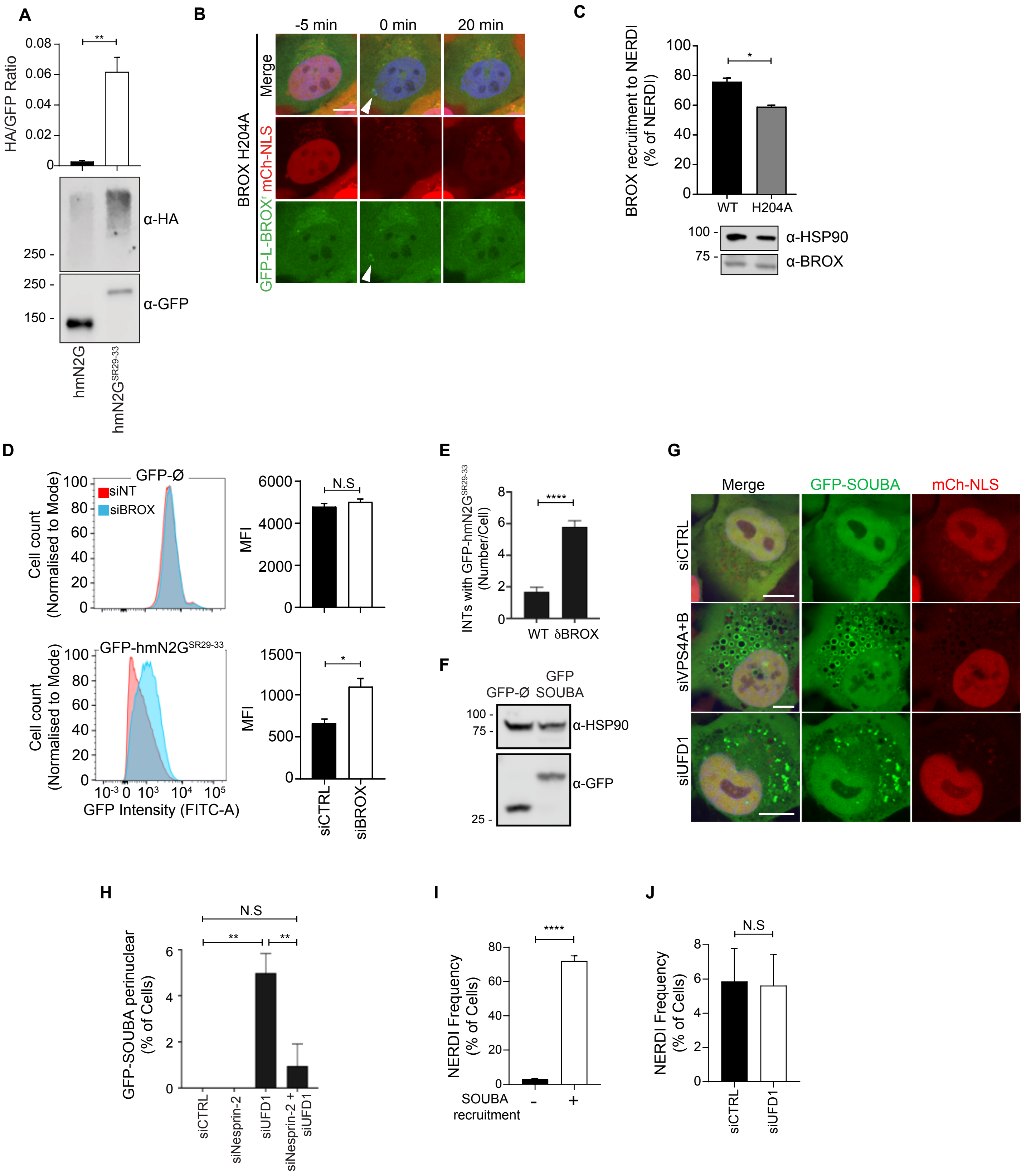

A
HA/GFP Ratio
0.08
0.06
0.04
0.02
0
**
α-HA
α-GFP
250 -
250 -
150 -
hmN2G
hmN2GSR29-33
B
-5 min
0 min
20 min
Merge
BROX H204A
GFP-L-BROXr mCh-NLS
C
BROX recruitment to NERDI (% of NERDI)
100
80
60
40
20
0
*
WT
H204A
100 -
75 -
α-HSP90
α-BROX
D
GFP-Ø
siNT
siBROX
Cell count (Normalised to Mode)
MFI
6000
4000
2000
N.S
GFP-hmN2GSR29-33
1500
1000
500
*
10-3 0 103 104 105
GFP Intensity (FITC-A)
siCTRL
siBROX
E
INTs with GFP-hmN2GSR29-33 (Number/Cell)
8
6
4
2
0
****
WT
δBROX
F
GFP-Ø
GFP SOUBA
100 -
75 -
25 -
α-HSP90
α-GFP
G
Merge
GFP-SOUBA
mCh-NLS
siCTRL
siVPS4A+B
siUFD1
H
GFP-SOUBA perinuclear (% of Cells)
N.S
**
**
siCTRL
siNesprin-2
siUFD1
siNesprin-2 + siUFD1
I
NERDI Frequency (% of Cells)
****
SOUBA recruitment
-
+
J
NERDI Frequency (% of Cells)
N.S
siCTRL
siUFD1

Figure S4: BROX-CHMP5 interaction is not required for BROX recruitment to rupture sites and GFP-SOUBA perinuclear rings form in cells that will undergo NERDI. Related to Figure 4.

(A) GFP pull-down experiments of 293T transiently co-expressing HA-Ubiquitin together with GFP-hmN2G$^{SR29\text{-}33}$ or GFP-hmN2G. Eluted fractions were examined by Western blot using the indicated antibodies and the ratio between HA and GFP bands intensity in the eluted fractions was plotted. p=0.0036

(B-C) Time-lapse analysis of HT1080 cells expressing mCherry-NLS and GFP-L-BROX$^{r.}$ wild-type (WT) or mutant (H204). Hoechst was added to stain DNA. (B) Representative images of the quantifications shown in (C). Scale bar, 5µm. Arrowheads highlight site of rupture (C) Percentage of BROX-decorated NERDI. WT n=38; H204A n=59 cells; p=0.0244. The movies used to quantify recruitment for WT conditions were the same as the ones used for the recruitment quantification shown in Figure 3D. Cell lysates were examined by blotting with the indicated antibodies.

(D) FACS analysis of HT1080 cells stably expressing GFP-empty (GFP-ø) or GFP-hmN2G$^{SR29\text{-}33}$ after transfection with indicated siRNAs. Left: GFP fluorescence intensity profiles corresponding to one representative experiment. Right: quantification of GFP mean fluorescence intensity (MFI). $^{*}p<0.05$, $^{N.S}p>0.05$.

(E) Quantitation of the number of INTs decorated with Nesprin-2G per cell in HT1080 and HT1080$^{\delta BROX}$ cells stably expressing GFP-hmN2G$^{SR29\text{-}33}$. WT n=16; δBROX n=10; p<0.0001.

(F) Analysis of GFP-SOUBA expression in HT1080 cells stably expressing GFP-ø or GFP-SOUBA. Cell lysates were analysed by blotting with indicated antibodies.

(G-J) Live imaging of HT1080 cells co-expressing GFP-SOUBA and mCherry-NLS. (G) Analysis of GFP-SOUBA localisation in cells transfected with the indicated siRNAs. Scale bars, 10µm. (H) percentage of cells with perinuclear GFP-SOUBA accumulation. siCTRL n=588; siNesprin2 n=437; siUFD1 n=504; siNesprin2+siUFD1 n=468; $^{**}p=0.025$, $^{N.S}p>0.05$. (I) Analysis of GFP-SOUBA perinuclear accumulation in cells undergoing NERDI. SOUBA-negative n=1857; SOUBA-positive n=88; p<0.0001. (J) Percentage of cells showing NERDI after transfection with indicated siRNA. siCTRL n=2016; siUFD1 n=1910, p=0.9309.
